# Chemical signatures of femoral pore secretions in two syntopic but reproductively isolated species of Galápagos land iguanas (*Conolophus marthae* and *C. subcristatus*)

**DOI:** 10.1038/s41598-020-71176-7

**Published:** 2020-08-31

**Authors:** Giuliano Colosimo, Gabriele Di Marco, Alessia D’Agostino, Angelo Gismondi, Carlos A. Vera, Glenn P. Gerber, Michele Scardi, Antonella Canini, Gabriele Gentile

**Affiliations:** 1 Institute for Conservation Research, San Diego Zoo Global, 15600 San Pasqual Valley Road, Escondido, CA 92027-7000 USA; 2 Department of Biology, University of Rome Tor Vergata, Via della Ricerca Scientifica, 00133 Rome, Italy; 3 Galápagos National Park Directorate, Technical Biodiversity Research, Av. C. Darwin, Puerto Ayora, 200350 Isla Santa Cruz, Galápagos, Ecuador

**Keywords:** Zoology, Herpetology, Evolutionary ecology, Chemical ecology

## Abstract

The only known population of *Conolophus marthae* (Reptilia, Iguanidae) and a population of *C. subcristatus* are syntopic on Wolf Volcano (Isabela Island, Galápagos). No gene flow occurs suggesting that effective reproductive isolating mechanisms exist between these two species. Chemical signature of femoral pore secretions is important for intra- and inter-specific chemical communication in squamates. As a first step towards testing the hypothesis that chemical signals could mediate reproductive isolation between *C. marthae* and *C. subcristatus*, we compared the chemical profiles of femoral gland exudate from adults caught on Wolf Volcano. We compared data from three different years and focused on two years in particular when femoral gland exudate was collected from adults during the reproductive season. Samples were processed using Gas Chromatography coupled with Mass Spectrometry (GC–MS). We identified over 100 different chemical compounds. Non-Metric Multidimensional Scaling (nMDS) was used to graphically represent the similarity among individuals based on their chemical profiles. Results from non-parametric statistical tests indicate that the separation between the two species is significant, suggesting that the chemical profile signatures of the two species may help prevent hybridization between *C. marthae* and *C. subcristatus*. Further investigation is needed to better resolve environmental influence and temporal reproductive patterns in determining the variation of biochemical profiles in both species.

## Introduction

Iguanas are among the most representative animal species of Galápagos Islands, one of the most paradigmatic locations for the development of evolutionary thinking. Three species of land iguanas occur on the islands and are endemic to the archipelago: *Conolophus subcristatus*, *C. pallidus*, and *C. marthae*. *Conolophus subcristatus* (Galápagos Land Iguanas or simply Yellow Land Iguanas) are widespread and currently distributed on the islands of Santa Cruz, Plaza Sur, Seymour Norte (introduced), Baltra (repatriated), Santiago (recently reintroduced), Isabela, and Fernandina. In contrast, *Conolophus pallidus* (Barrington Land Iguanas) are limited to Santa Fe Island, and *C. marthae* (Galápagos Pink Land Iguanas or simply Pink Iguanas), a recently described species^1,2^, are limited to the northern slopes of Wolf Volcano (WV hereafter) on Isabela Island. Pink iguanas are currently listed as Critically Endangered in the IUCN Red List^3^. Only a single population of *C. marthae* exists, in syntopy (sensu Rivas^4^) with a much larger population of *C. subcristatus*.

Land iguanas are evolutionarily related to marine iguanas (*Amblyrhynchus cristatus*). The two genera are sister taxa and started diverging about 4.5 Ma^5^. Marine iguanas, also endemic to Galápagos, are distributed across all major islands of the archipelago and many islets. Despite their evolutionary divergence, morphological differentiation and ecological separation, *A. cristatus* and *C. subcristatus* may occasionally hybridize and generate viable, yet non-fertile, offspring^6^. Interestingly, no hybridization between *C. marthae* and *C. subcristatus* has currently been found, although the two species could have hybridized in the past^2,7^. This is peculiar because, as *A. cristatus* and *C. subcristatus* in Plaza Sur testify, squamate lizards seem particularly prone to hybridization not only between closely related species but also between genetically divergent taxa^8,9^. The apparent lack of hybridization between *C. marthae* and *C. subcristatus* suggests the existence of effective reproductive isolating mechanisms (RIMs), although it is not possible to completely exclude postzygotic RIMs. Past hybridization between the two species might have enhanced the evolution of precopulatory RIMs by reinforcement^7,10,11^.

Pheromonal communication is known in squamate reptiles where social behaviour may be chemically mediated^12,13^, and differentiation in chemical signals, within a reproductive context, can act as a precopulatory RIM^9^. In particular, chemicals released during the breeding season can foster mate recognition in different reptiles, thus preventing gene flow between closely related and/or syntopic species^14–16^. Chemical recognition as a possible way to prevent interspecific hybridization is known in different species of lizards. For example, chemical cues may be involved in preventing hybridization between the endemic insular *Podarcis atrata* and the invasive *Podarcis hispanica*^17,18^. Despite the growing interest around chemical communication in reptiles, studying the chemical ecology of some species within this group remains challenging. This is particularly true when working with critically endangered species that can only be found in remote areas of the world, and for which behavioural trials and experiments are logistically complicated. In such cases, describing their chemical profile and highlighting statistical differences can be a good starting point^13^. An extensive literature search revealed that, despite being iconic animals, Galápagos iguanas have received little attention regarding the analysis of chemical compounds and their potential role for intra- and inter-specific communication. The first milestone on the path to fill this knowledge gap was recently reached by Ibáñez and colleagues who described differences in chemical profiles from femoral pore secretions collected across multiple populations of marine iguanas^19^. In iguanine lizards, femoral glands are the organs largely involved in chemical communication^20,21^. These glands are epidermal structures located on the ventral surface of the hind legs, close to the pre-cloacal abdominal area. Ibáñez and colleagues found that certain molecules can play a significant role in intra-specific chemical communication, and even correlate with certain morphological characteristics^19^.

In this paper, we describe the chemical compound diversity in femoral pore secretions of Galápagos land iguanas in the genus *Conolophus*. We specifically focus on the only known population of critically endangered *C. marthae* and its syntopic congener, *C. subcristatus*. Due to the lack of documented hybridization between these species, we hypothesized that chemical cues could contribute to reproductive isolation. As a first step towards describing chemically mediated reproductive isolating mechanisms, we tested the prediction that differences in femoral pore secretions exist between the two species and discuss the possible role of chemical compounds in preventing admixture and hybridization between *C. marthae* and *C. subcristatus*.

## Results

Our final data-set was composed of 227 sampled individuals: 150 from 2012 (78 *C. marthae* and 72 *C. subcristatus*), 60 from 2014 (30 *C. marthae* and 30 *C. subcristatus*), and 17 from 2015 (8 *C. marthae* and 9 *C. subcristatus*; Table 1). In total, 113 different molecular compounds were identified by GC–MS analysis of samples collected for this study: 111 in 2012, 96 in 2014, and 94 in 2015 (see Table 1). When considering the entire data-set, none of the chemical compounds identified were unique to either species. However, examination of the dataset by year, species and sex revealed differences in the presence/absence of chemical compounds between species and/or between sexes within species (a complete description of all molecules distinguishing species by year and sex is available in the Supplementary Materials and Supplementary Tables S1–S6).

**Table 1:** Number of femoral pore samples collected and analyzed for adult *Conolophus marthae* (pink) and *C. subcristatus* (yellow) iguanas.

		
	N	R_chem_	H_chem_	N	R_chem_	H_chem_
**2012 (rs)**						
F	24	111	2.97 (± 0.33)	31	109	2.88 (± 0.37)
M	54	111	3.11 (± 0.26)	41	111	3.05 (± 0.36)
**2014 (rs)**						
F	15	82	2.79 (± 0.17)	13	84	2.66 (± 0.24)
M	15	78	2.81 (± 0.14)	17	87	2.74 (± 0.30)
**2015 (nrs)**						
F	3	76	3.14 (± 0.12)	0	NA	NA
M	5	82	2.98 (± 0.10)	9	90	3.07 (± 0.22)

Samples are divided by year of capture and sex (F = Females, M = Males). In 2012 and 2014 samples were collected during the reproductive season (rs), whereas in 2015 they were collected during the non-reproductive season (nrs). This table also shows the number of different lipophilic compounds identified by GC–MS in femoral pore secretions (R_*chem*_), and the mean and standard deviation of chemical diversity (H_*chem*_).

**Figure 1:**
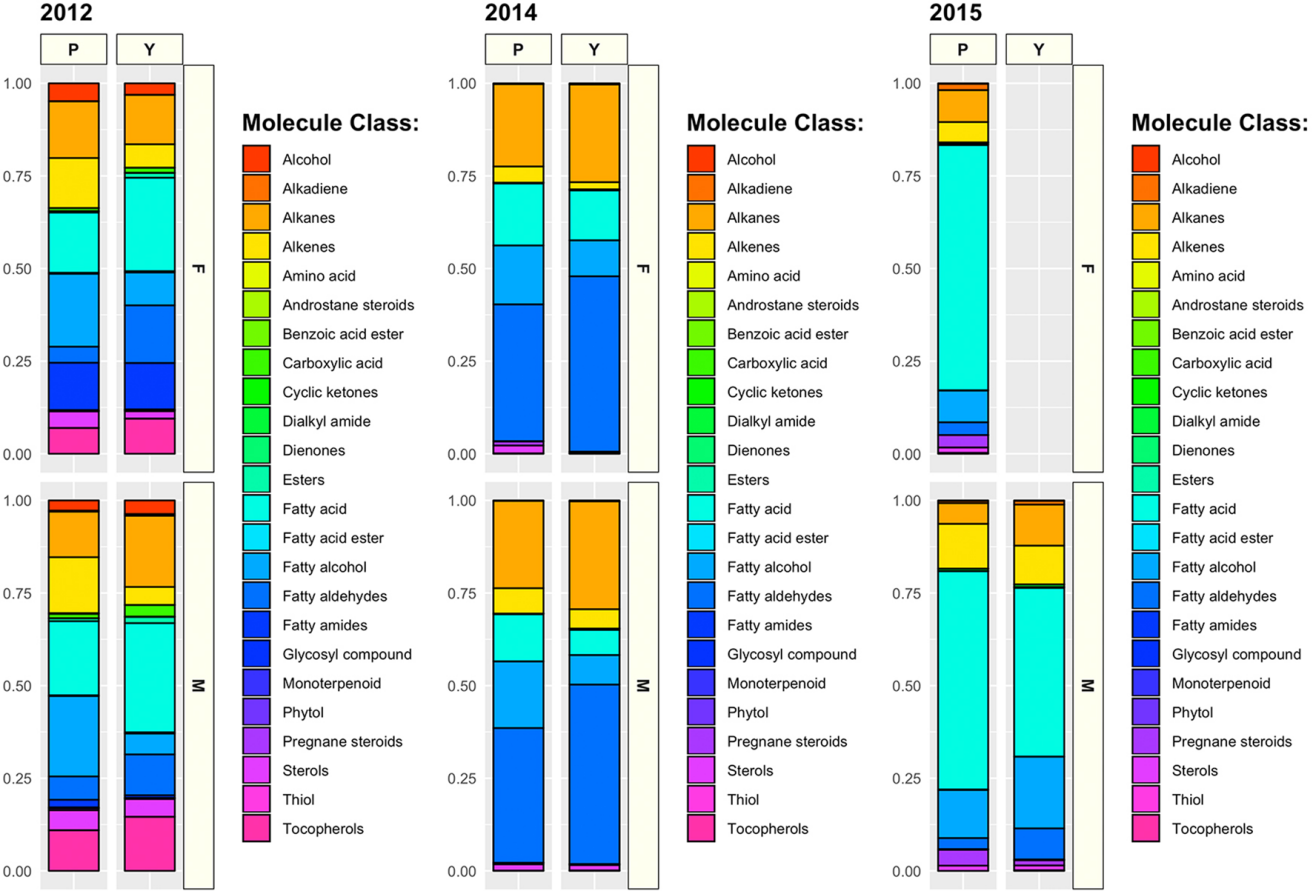
Relative abundance of different compounds by molecular class found across three years of sampling. Data are organized by species (P = *C. marthae*; Y = *C. subcristatus*) and sex (M = Males; F = Females).

The relative abundance of different compound classes varied across years and correlated with the number of individuals sampled, with samples collected in 2012 (n = 150) presenting the highest values of chemical richness (109 ≤ R_*chem*_ ≤ 111; see also Table 1 and Fig. 1). Despite being collected during the reproductive season, samples from 2014 (n = 60) showed values of chemical richness more similar to those collected in 2015 during the non-reproductive season (n = 17; Table 1).

**Figure 2:**
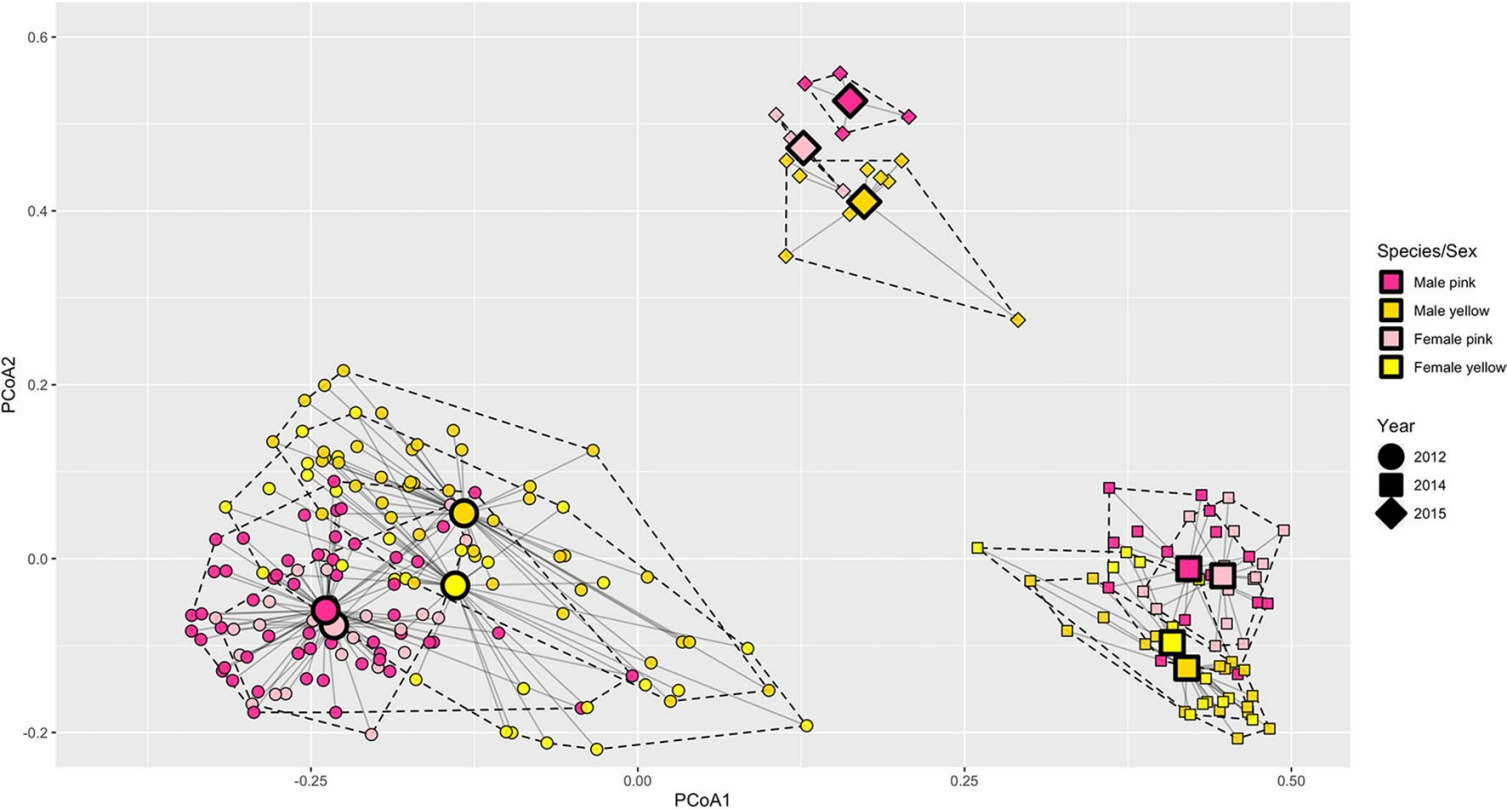
Principal coordinate analysis approximating the multivariate homogeneity of groups variance. We used the Bray–Curtis dissimilarity index. The graph shows how much the data points (smaller symbols), analyzed by year, species and sex, disperse from a group centroid (larger symbols) representing the mean value calculated across the multivariate space. The first axis (PCoA1) explains 18.23% of the total variance, while the second axis (PCoA2) explains 5.84% of the total variance. The organization of points in the multivariate space suggests a deep differentiation between sampling years.

Individuals of the two species clustered separately in the non-metric multidimensional scaling (nMDS) scatterplot across sampling years (Supplementary Figure S1). The analysis of multivariate spread in our data also shows a clear separation between samples collected in 2012, 2014 and 2015 (Fig. 2). Based on this evidence and because the assumption of equal multivariate spread was upheld in 65% of the Tukey’s HSD pairwise comparisons, (Supplementary Figure S2) we proceeded with the permutational multivariate analysis of variance (PERMANOVA). In fact, we do not expect that the slight difference in multivariate spread observed might strongly contribute to the effects detected by the analysis. PERMANOVA confirmed that observed differences in chemical composition between species across years were significant (p_Year x Species_ << 0.001; Table 2). We also found a significant effect of sex (p_Sex_ << 0.001; Table 2) and a significant interaction between year, species, and sex (p_Year x Species x Sex_ = 0.020; Table 2), indicating probable differences in the production of chemical compounds between males and females of the same species across years.

**Table 2:** Results of permutational multivariate analysis of variance (PERMANOVA) showing degrees of freedom (DF), sequential sum of squares (SumOfSqs), partial R squared values (R^2^), F statistics (F), and *p* values based on N permutations (P (> F)) where N = 9,999.

		Df		SumOfSqs		R^2^		F	P (> F)
Year		1		14.51		0.26		92.94	<< 0.001
Species		1		2.67		0.04		17.14	<< 0.001
Sex		1		1.09		0.02		6.97	<< 0.001
Year x Species		1		1.89		0.03		12.1	<< 0.001
Year x Sex		1		0.94		0.01		6.05	<< 0.001
Species x Sex		1		0.31		0.00		1.99	0.052
Year x Species x Sex		1		0.39		0.00		2.50	0.020
Residual		219		34.18		0.51			
Total		226		55.94		1.00			

The similarity percentage analysis (SIMPER) indicated numerous molecules contributing to species differentiation within the reproductive seasons of 2012 and 2014 (Supplementary Figure S3). Of these, a group of seven molecules were consistently driving the pattern of differentiation between the two species across seasons (Table 3). Moreover, the relative abundance of these seven molecules varied dramatically over the two reproductive seasons (Supplementary Figure S4) contributing to the detected variation between them. A principal component analysis using these molecules revealed that 10-Henicosene was consistently explaining the largest amount of variance in our samples (> 30% of variance explained, Supplementary Figures S5 and S6).

**Table 3:** List of molecules, after SIMPER analysis, consistently contributing to species differentiation across the two reproductive seasons (*p* < 0.01).

Class	Compound name	Chem ID
Fatty alcohol	n-Tridecanol	8207
Fatty alcohol	2-Hepten-4-ol	5366235
Alkenes	10-Henicosene	5364553
Fatty acid	Heptadecyl heptadecanoate	10465
Fatty acid	10-Heptadecenoic acid	6029464
Fatty acid	11-Hexadecenoic acid	5364677
Fatty acid	Hexadecenoic acid	5363255

Compound names and reference numbers (Chem ID) for the on-line PubChem database are listed. Results from SIMPER were not statistically significant for compounds with Chem IDs 5366235, 10465, and 5364677 after applying a Bonferroni test and adjusting the* p* value (α = 0.05/113) to account for multiple comparisons.

**Figure 3:**
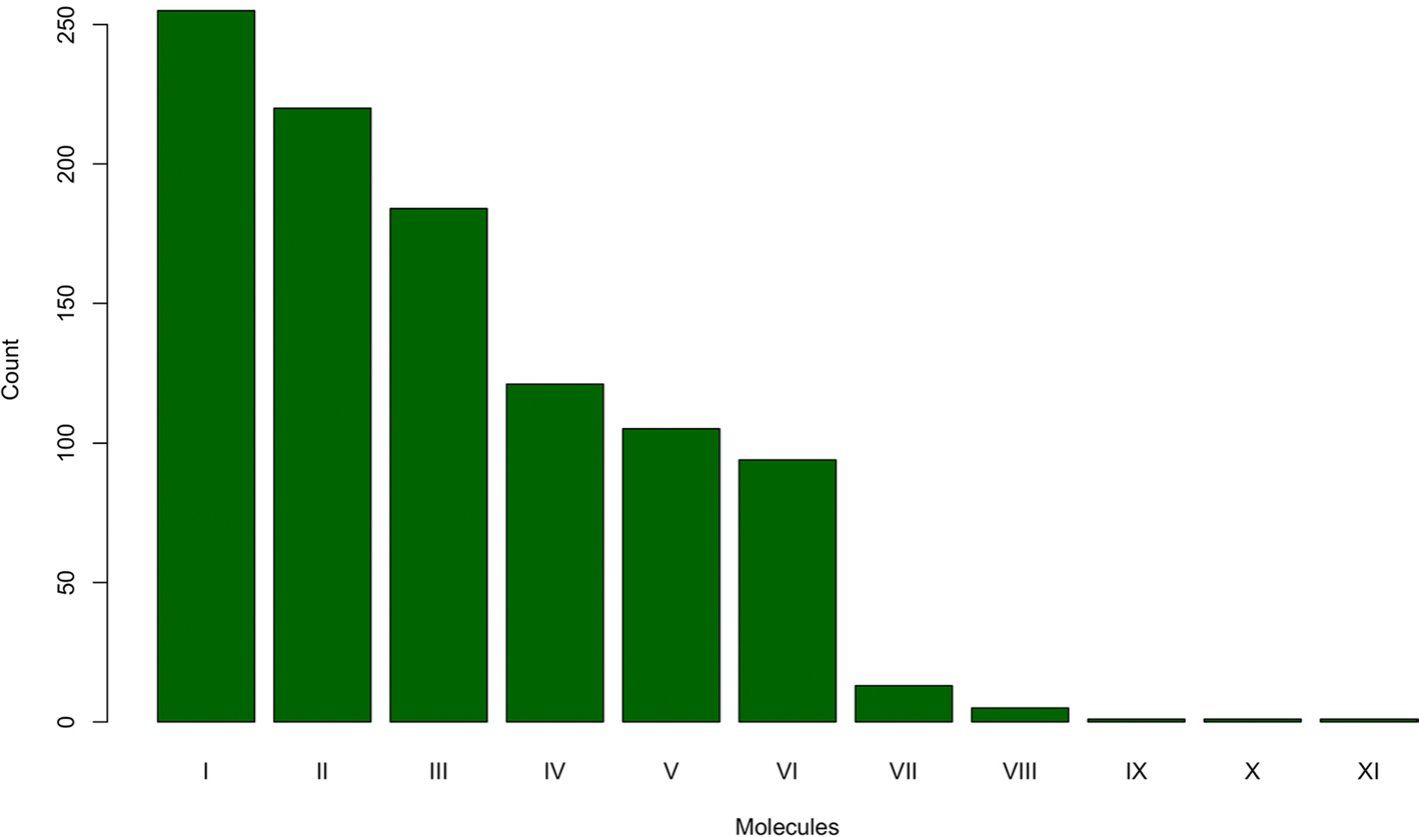
We recorded the most important chemical compound identified in each of the 1,000 independent random forest models (relative importance is based on the Gini Index). The bar plot shows the frequency that each of 11 compounds identified as most important: **I**: 1-Heneicosanol (Fatty alcohol); **II**: 10-Henicosene (Alkene); **III**: Hexadecenoic acid (Fatty acid); **IV**: Oleyl Alcohol (Fatty alcohol);**V**: 1-Hexadecanol (Fatty alcohol); **VI**: Cholestanol (Sterols); **VII**: Butenoic acid (Fatty acid); **VIII**: n-Tridecanol (Fatty alcohol); **IX**: 17-Hydroxypregnenolone (Pregnane steroids); **X**: Pregn-4-ene-3,20-dione (Pregnane steroids); **XI**: Stigmasterol (Sterols). A χ^2^ test indicated that we can reject the null hypothesis of each molecule having the same probability of being picked (χ^2^ = 1,001.8, df = 10, *p* << 0.001).

The random forest (RF) tuning algorithm indicated that 500 trees and 17 random variables at each split would produce the highest accuracy in the model (Supplementary Fig. 7). Based on this output we grew 500 trees in each forest using 17 random variables at each branching step. This procedure was repeated in all 1,000 randomized datasets. A group of only 11 molecules constantly received the highest Gini score (Fig. 3). Of these, 10-Henicosene, and n-Tridecanol were also identified in the SIMPER analysis. We performed a χ^2^ test to investigate whether there were statistically significant differences in the frequency with which these compounds had been picked by the RF algorithm, and the null hypothesis was rejected (*p* << 0.001). The average accuracy of our classification model estimated on the 1,000 randomized datasets was as high as 96.3% and we also found a very high and significant level of agreement between predicted and observed species assignment (Cohen’s Kappa = 0.924, *p* < < 0.001, Table 4). Furthermore, our model performed well at discriminating between true positive cases (i.e., at assigning pink iguanas to the appropriate classification group; Sensitivity = 96.6%, Table 4) and at identifying true negative cases (i.e., at assigning non-pink iguanas to the appropriate classification group; Specificity = 95.8%, Table 4). To better visualize the diagnostic ability of our model we built a Receiving-Operating-Characteristic (ROC) curve and calculated the area under this curve using the **evalm()** function from the *MLeval* R-package^22,23^. The area under the ROC curve is estimated at 96% of the sensitivity space (True Positive Rate) indicating that our classification model performs significantly better than the random assignment of individuals to the two different classes (see Supplementary Fig. 8).

**Table 4:** Summary of the average output of the Random Forest model runs validated using 1,000 datasets generated by randomly sampling the original dataset showing accuracy of the model (Accuracy), Cohen’s K, Lower and Upper 95% confidence interval (CI), a measure of the proportion of positive samples correctly identified (Sensitivity), a measure of the proportion of negative samples correctly identified (Specificity), and* p* value (*p*).

Index	Average
Accuracy	0.963
Cohen’s K	0.924
Lower 95% CI	0.907
Upper 95% CI	0.990
Sensitivity	0.966
Specificity	0.958
*p*	< < 0.001

## Discussion

In this study, we describe the chemical variability of femoral pore secretions extracted from two species of land iguanas in Galápagos. We found an incredibly variable array of chemical compounds. Ibáñez and colleagues reported 20 different lipids isolated from samples collected from three individuals of *C. subcristatus* from an outdoor enclosure at the Charles Darwin Station on Santa Cruz Island, Galápagos^19^. Here we report over 100 different molecules. Other authors have described the astonishing chemical complexity and diversity in lizards using a variety of analytical approaches, from thin-layer chromatography (TLC^13^) to polyacrylamide gel electrophoresis^24^, to gas-chromatography coupled with mass spectrometry (GC–MS^14,19^). The high sensitivity of the GC–MS protocol used in this study^25^, coupled with the large number of individuals analyzed, can explain the high chemical complexity we observed. Nevertheless, it is worth noting that the complexity and variety of chemical signals that animal species can detect, greatly outperforms the sensitivity of current GC–MS methodologies^26^, and we may have underestimated the actual complexity of the chemical signature of these species.

Only 10 of the 20 molecules identified from *C. subcristatus* in Ibáñez et al.^19^ were also found in the current study (Octadecanoic acid, Hexadecanal, Octadecanal, Eicosanoic acid, 10-Hexadecanoic acid, 11-Octadecenoic acid, 9-Hexadecenoic acid, Cholesterol, Cholestanol, and 12-Docosanoic acid). This is not surprising, though, considering that the samples analyzed by Ibáñez and colleagues were collected during a different period of the year and were from captive animals belonging to a different island population. Some authors have described how chemical production and composition of femoral gland secretions in iguanid lizards can vary seasonally^27^. Other authors have reported how conspecific individuals from different populations may sometimes be characterized by different signatures in their chemical profiles^28,29^. Finally, it has been reported how intraspecific variability among individuals can also be very high^30^.

We observed a high degree of similarity between chemical signatures of the two syntopic species. However, reptiles can discriminate between even subtle differences in chemical profiles. It is interesting to note that in many reptile species even minor differences in certain chemical compounds can be associated with different responses in conspecific or heterospecific individuals, not necessarily within a reproductive isolation context. For example, males of red-sided garter snakes can assess body length and body condition of potential mates using only chemical cues, including the relative amount of specific chemical compounds^31^. A study of *Sceloporus graciosus* lizards showed that differences in the amount of certain chemical compounds may be associated with mating decisions and territorial behavior^32^. Taken together, these data suggest that even though the chemical profiles of *C. marthae* and *C. subcristatus* are similar in composition, individuals behavioural response may be guided by even small differences in the relative amount of compounds.

The similarity in chemical profiles may depend on more than one factor and, in our case, we see two likely non-mutually exclusive explanations. First, it has been shown how different species of lacertids sharing the same type of environment are more likely to produce the same type of compounds^16^. Second, although non-definitive, there is evidence suggesting that certain dietary traits may influence the type of chemicals produced in glandular secretions^13,33,34^. A combination of these factors could explain our results because *Conolophus marthae* and *C. subcristatus* are syntopic and during the reproductive season adults of both species gather around the caldera of Wolf Volcano. It is therefore plausible that, at least during this time, their diet partly overlaps. It is also worth noting that resource availability on top of the volcano is largely dependent on the intensity of the rainy season, and may influence the type of vegetation available for foraging^35^. Therefore, yearly differences in food availability could potentially affect the production and concentration of different chemical components in the two species. Outside of the reproductive season, iguanas seem to scatter along the slopes of the volcano, where different environmental conditions and trophic resources exist^36^. This could be reflected in the results of PCoA in which the second axis separated the 2015 samples (collected during the non-reproductive season) from the 2012 and 2014 samples (collected during the reproductive season), which were not distinguishable along the axis. Dispersion during the non-reproductive season could provide access to very different trophic resources, and diets of the species might diverge more substantially during this time. More detailed data on temporal habitat use by the two species is needed to fully test this hypothesis.

Our data are not definitive but suggest a possible role for chemical communication in reinforcing reproductive isolating mechanisms between the two land iguana species. Our statistical analysis using RF showed that certain molecules are very good species classifiers. In particular, 1-Heneicosanol and 10-Henicosene are excellent molecules for differentiating the two species. Fatty-alcohols (like 1-Henicosanol) are used by males of the lacertid lizard *Acanthodactylus boskianus* to recognize intraspecific males and avoid them^37^. N-Alkenes have been shown to play an important role in intraspecific recognition in other taxa^38,39^, but their role in reptiles has yet to be investigated. In other lizards, a size-dependent chemosensory response for intraspecific recognition has been described^40^. It is therefore plausible to speculate on the possible role of some of these molecules for intraspecific recognition, although we recognize that differences in chemical profiles may not always be involved in reproductive isolation^29,41^. In addition to these two molecules, our machine learning algorithm identified 9 additional molecules that are significantly important in distinguishing between *C. marthae* and *C. subcristatus* (Fig. 3). We can combine the information presented in this paper with other studies on the evolution and ecology of these two land iguana species. *Conolophus marthae* and *C. subcristatus* are thought to have diverged around 1.5 Ma^5^. This estimate is much more recent when compared to the divergence time of other iguana species and genera (e.g., *Conolophus*/*Amblyrhynchus* estimated at 4.5 Ma; *Iguana*/*Cyclura* estimated at 12 Ma ^5^). Yet, evidence for extensive recombination between even distantly related iguanid lizards exists^6,9,42^. Indeed, despite their relatively recent divergence, *C. marthae* and *C. subcristatus* are morphologically very distinct. Moreover, the display action pattern of the two species (commonly known as head-bobbing behavior and shown to be important in communication among conspecific iguanid species^43^) is very different^1^. In *Conolophus*, head-bobbing behaviour is performed for territory defence and, with slightly different patterns, within a reproductive context. Additionally, the two species seem to have different ecological requirements^36^. Thus, while several factors may contribute to preventing hybridization between the two species, with the present study, we provide preliminary evidence consistent with a hypothesis that certain molecules play a role in maintaining reproductive isolation between *C. marthae* and *C. subcristatus*. Further studies and targeted behavioural experiments are needed to tease out the potential role of specific molecules in the reproductive behaviour of these iguanas.

## Materials and methods

### Sample collection

We analyzed the chemical profile and composition of femoral pore secretions collected from *C. marthae* and *C. subcristatus* iguanas from WV on Isabela Island, Galápagos. Samples were collected during the reproductive season (i.e., late May-early July) in 2012 and 2014. In addition, we opportunistically collected samples from a small number of individuals outside the reproductive season (i.e., November) in 2015. Upon capture, according to a protocol approved by the Galápagos National Park, the femoral secretion was obtained by gently squeezing the femoral glands of iguanas. Secretions were extracted using sterile metal tweezers. Between sampling of different individuals, tweezers were cleaned with 90% ethanol. Plugs associated with secretions from large pores were removed and discarded. Samples were stored in 2 ml sterile cryotubes and maintained at −10 °C while in the field. They were subsequently transported at − 78 °C (dry ice) to the laboratory, where they were processed.

### Sample preparation and GC–MS analysis

Femoral gland secretions were processed following Escobar et al*.*^44^, with some modifications. Briefly, 10 mg of sample was dissolved in 600 µl of dichloromethane and 600 µl of ethyl acetate and centrifuged for 1 h at room temperature. Then, after centrifugation for 10 min at 14,000 rpm, the supernatant was recovered and preserved. The pellet was then used to repeat the digestion and centrifugation steps a second time. The supernatant fractions obtained were mixed and dried out using a speed-vac system (Eppendorf AG 22,331 Hamburg, Concentration Plus). Finally, the lyophilized sample was re-suspended with 100 µl of dichloromethane derivatized with 100 µl of Methyl-8-Reagent (Thermo Fisher Scientific ©), according to the manufacturer’s guidelines, and subjected to chromatographic analysis using a GC–MS instrument (QP2010 Shimadzu, Japan). Chromatographic elution of the samples (2 µl) was performed using a DB-5 column (30 m × 0.25 mm × 0.25 µm; Agilent Technologies, Santa Clara, CA, USA) and setting the GC oven as follows: 70 °C for 2 min, then increased 9 °C/min up to 280 °C and held isothermally for 18 min, with a 43 min and 3-s long run. Helium was used as a carrier gas at a constant flow of 2.1 ml/min. MS conditions and details about the identification and quantitation (as a percentage of relative abundance) of the molecules was carried out following Gismondi et al*.*^25^ and Giovannini et al*.*^45^. Only molecules with a similarity index higher than 85% were retained in the analysis.

### Statistical analysis

#### Chemical diversity

To investigate general differences in chemical production we first calculated the total number of different compounds found in the two iguana species (chemical richness, R_chem_). We also calculated an index of chemical diversity for each individual using the formula H_chem_ =  − ∑*p*_*i*_ * ln *p*_*i*_, where *p*_*i*_is the relative abundance of the *i*th molecule in each sample^30,46^.

We grouped individuals based on ranking (i.e., non-metric distances) of their chemical profiles using non-metric multidimensional scaling (nMDS). This ordination technique makes few assumptions about the data and their distribution. We used the Bray–Curtis dissimilarity index as used in Ibáñez et al*.*^19^ to calculate a matrix of the compositional chemical dissimilarity profiles between individuals. We then plotted individual chemical profiles along X–Y–Z axes. Points that are closer together represent individuals with more similar chemical profiles than those further away (see also Mason and Parker^12^). To assess significant differences in the chemical composition and relative abundance of compounds between species we used a non-parametric multivariate statistical test based on permutations (PERMANOVA). The methodology assumes homogeneity of the multivariate variance spread in our data. This assumption is hardly met when dealing with large data sets, as we confirmed after testing for homogenity using the **betadisper()** function^47,48^, which implements Anderson's PERMDISP2 procedure for the analysis of multivariate homogeneity of group dispersions^49^. To determine the nature of the difference between any pair of groups we performed a nMDS and a Principal Coordinate Analysis, in agreement with Anderson’s recommendation in the PERMANOVA user notes. In addition, following the approach suggested by Oksanen and collaborators^47^, we performed Tukey’s HSD tests to determine the percentage of pairwise case-comparisions in which distances from centroids were statistically different. We then tested for significance in multivariate mean differences using 9,999 permutations with the **adonis2()** function from the *vegan* package^48^ in R v.3.5^23^. We used “species”, “sex” and “year of collection” as fixed effects, and also tested for interactions between these factors. The different strata of our experimental design (i.e., three sampling years, each with four treatments—males and females of two different species) were accounted for using the option ***strata***^48^.

To identify compounds differentiating the two species and potentially underlying a reproductive isolating mechanism, we focused our analysis on samples collected during the reproductive season (i.e., samples collected in 2012 and 2014). We used a similarity percentage analysis (function **simper()** within the *vegan* package^39^). The algorithm is based on the decomposition of the Bray–Curtis dissimilarity index and returns the most important variables in driving dissimilarity patterns^48^. We ran the algorithm on samples from 2012 and 2014 separately and looked for those molecules that consistently drove the pattern of dissimilarity between species across years. The identified molecules were then used in a principal component analysis (PCA). This analysis allowed the identification of variables associated with the highest variance and accounted for collinearity in our data. We selected molecules explaining up to 75% of the total variance. All statistical calculations and analyses were run in R v.3.5^23^.

#### Random forest

We also analyzed the problem of species differentiation from a binary classification perspective. If chemical cues (chemical composition and/or abundance of specific compounds) are potentially involved in intraspecific recognition, we can then ask the following question in statistical terms: what chemical features are most important for species classification? To answer this question, we used a machine-learning algorithm, specifically a Random Forest (RF). Random Forest is a type of supervised classification algorithm where a group of individuals (observations) is divided into subgroups using different variables (classifiers). The RF algorithm offers a series of advantages over multivariate statistical analyses. First, it can handle large multivariate datasets. Second, the supervised algorithm creates multiple classification trees and combines them into an ensemble model by majority voting. Third, the randomization of the variables used to build suboptimal trees avoids overfitting issues, thus making the resulting ensemble, i.e. the RF, a stronger learner and a better model. Finally, the algorithm can be tuned to find an optimal set of parameters to improve its performance^50^. We ran the RF algorithm on the entire dataset of *C. marthae* and *C. subcristatus* samples collected across all years. We used “species” as a categorical response variable and the identified chemicals as explanatory variables (or classifiers). The complete dataset was divided into “training” and “test” datasets with approximately 50% of observations in the training dataset and the remainder in the test dataset. This was achieved by randomly sampling individuals from the original pool. The proportion of *C. marthae* and *C. subcristatus* samples in training and test datasets were kept similar to their respective proportions in the original dataset. The training dataset is used to split observations in to subgroups and to evaluate the performance of each classifier. The test dataset is then used to validate the result and estimate overall accuracy of the model^50^. Because of the stochastic nature of this process, we repeated this procedure independently 1,000 times (i.e., we created 1,000 independent randomly selected datasets and ran the RF algorithm on all of these datasets). The algorithm grows multiple decision trees. In each tree, a classification is made so that at each node of the growing tree only a random subset of the explanatory variables (i.e., molecules) is used to split the parent node into the child nodes (i.e., to establish whether an individual should be classified as *C. marthae* or *C. subcristatus*). At the end of the algorithm, we looked at the “forest” composed of these multiple “trees” (models) and selected the variables that contributed the most throughout the splitting process. The number of trees to grow in each forest and the number of explanatory variables to be used was established using an algorithm-tuning procedure. We used the Gini Index^51^ to determine how well the variables were performing during their splitting process. The overall accuracy of the model was obtained by averaging the accuracy of all independent models. This procedure was implemented in R v.3.5^23^ using the *randomForest* and *caret* packages^52,53^ (model details are available in the supplementary materials and the R code is available upon request).

### Ethical approval

Animal manipulation and sampling were performed according to a protocol that minimized animal stress, following the European Community guidelines and with the approval of the Galápagos National Park Directorate (GNPD). The GNPD does not have a specific ethics committee. However, it is responsible for the administration of research (including issuing research permits) carried out in the Galapagos Protected Areas, in its capacity as administrator of these areas and representative of the National Environmental Authority. The GNPD granted a research permit to G.G. for this project. Samples were exported and imported under GNPD and CITES permits issued to G.G.

## Supplementary information

Supplementary file1
