# Supplementary material for: Chemical signatures of femoral pore secretions in two syntopic but reproductively isolated species of Galápagos land iguanas (Conolophus marthae and C. subcristatus)
Source: Sci Rep. 2020 Aug 31;10:14314. doi: 10.1038/s41598-020-71176-7 (PMC7458923; doi:10.1038/s41598-020-71176-7)
Supplement: Supplementary file 1 — Supplementary file1 [file 41598_2020_71176_MOESM1_ESM.pdf]

Chemical signatures of femoral pore secretions in two syntopic but reproductively isolated species of Galápagos land iguanas.

Giuliano Colosimo[1,2], Gabriele Di Marco[2], Alessia D'Agostino[2], Angelo Gismondi[2], Carlos A. Vera[3], Glenn P. Gerber[1], Michele Scardi[2], Antonella Canini[2], and Gabriele Gentile[2, *]

[1]1Institute for Conservation Research, San Diego Zoo Global, 15600 San Pasqual Valley Road, Escondido CA 92027-7000 USA
[2]2Department of Biology, University of Rome Tor Vergata, Via della Ricerca Scientifica - 00133 Rome, Italy
[3]Galápagos National Park Directorate, Technical Biodiversity Research, Av. C. Darwin, Puerto Ayora 200350 Isla Santa Cruz, Galápagos, Ecuador
[*]Corresponding author: gabriele.gentile@uniroma2.it

ABSTRACT

The only known population of *Conolophus marthae* (Reptilia, Iguanidae) and a population of *C. subcristatus* are syntopic on Wolf Volcano (Isabela Island, Galápagos). No gene flow occurs suggesting that effective reproductive isolating mechanisms exist between these two species. Chemical signature of femoral pore secretions is important for intra- and inter-specific chemical communication in squamates. As a first step towards testing the hypothesis that chemical signals could mediate reproductive isolation between *C. marthae* and *C. subcristatus*, we compared the chemical profiles of femoral gland exudate from adults caught on Wolf Volcano. We compared data from three different years but focused on two years in particular when femoral gland exudate was collected from adults during the reproductive season. Samples were processed using Gas Chromatography coupled with Mass Spectrometry (GC-MS). We identified over 100 different chemical compounds. Non-Metric Multidimensional Scaling (nMDS) was used to graphically represent the similarity among individuals based on their chemical profiles. Results from non-parametric statistical tests indicate that the separation between the two species is significant, suggesting that the chemical profile signatures of the two species may help prevent hybridization between *C. marthae* and *C. subcristatus*. Further investigation is needed to better resolve environmental influence and temporal reproductive patterns in determining the variation of biochemical profiles in both species.

Supplementary Materials

Supplementary Materials and Methods

Random Forest

Random Forest is a type of supervised classification algorithm where a group of individuals (observations) is divided into subgroups using different variables (classifiers). Multiple trees are grown from their roots (the initial group of observations) and they split into branches separating the group into two (or more) categories. At the end of the algorithm (when we have a full grown forest) it is possible to identify the classifiers that performed best at creating splits (branches or subgroups) while maximizing similarities among observations within subgroups.

In our study the final output of our classification problem is categorical (i.e., the 2 species of iguanas, *C. marthae* or *C. subcristatus*). The molecules identified in the study become the classifiers. Our model is able to classify iguanas in the two different species based on their chemical signature.

The complete dataset was divided into training and test datasets with approximately 50% of observations in the training dataset and the remainder in the test dataset. The training dataset is used to split observation in to subgroups and to evaluate the performance of each classifier. The test dataset is then used to validate the result and estimate overall accuracy of the model.

To maximize the performance of the machine learning algorithm we searched for a combination of parameters that could help improve the accuracy of our model. In particular, we focused on the number of trees to grow in each forest and on the minimum number of random classifiers to split samples into subsamples (see Supplementary Figure S7).

We repeated this algorithm independently 1000 times and averaged the overall accuracy of the model. All code to process the dataset and perform calculation was written in R and can be released by the authors upon reasonable request.

Supplementary Results

For samples collected in 2012, all compounds found in *C. marthae* were also present in *C. subcristatus*. Examining differences between sexes within species, no compound was unique to either sex for *C. marthae*. However, for samples collected from *C. subcristatus*, two compounds found in males were not present in females: 8,11,14-Docosatrienoic acid, and Tetradecene.

In 2014, Methyl 18-methylnonadecanoate, 9-Tetradecenoic acid, Pregan-20-one, and Ergost-5-en-3-ol were present in samples from *C. marthae* but not *C. subcristatus*. In contrast, 3-Octadecene, Pentadecanoic acid, Eicosanoic acid, 2-Ethylhexyl octanoate, Campesterol, and 17-Hydroxypregnenolone were present in samples from *C. subcristatus* but not *C. marthae*. When comparing sexes within species, the following compounds were found in samples from male but not female *C. marthae*: Pentadecanal-, 9-Hexadecenoic acid, Erucylamide, Methyl hexacosanoate, 9-Tetradecenoic acid, Octadecedien-1-ol, Pregan-20-one, 2-Dodecenoic acid, whether Octadecanoic acid, 1-Tetradecanol, 9-Icosene, Methyl 14-methylpentadecanoate, Ethyl docosanoate, 8,11,14-Docosatrienoic acid, Methyl 18-methylnonadecanoate, Androsta-4,16-dien-3-one, Elaidyl alcohol, Cholesterol, and Ergost-5-en-3-ol. In contrast, 12-Docosanoic acid was found in samples from female but not male *C. marthae*. The following compounds were found in samples from male but not female *C. subcristatus*: 1-Tridecene, Tetradecane, Pentadecanal-, 9-Icosene, 1,16-Hexadecanediol, Elaidyl alcohol, Campesterol, and Octadecedien-1-ol. In contrast, Pentadecanoic acid, Eicosanoic acid, Methyl hexa-cosanoate, 1,19-Eicosadiene, and 2-Dodecenoic acid were present in samples from female but not male *C. subcristatus*.

In 2015, 1-Tridecene, Oleyl Alcohol, Cholest-4-en-3-one, and Campesterol were present in samples from *C. marthae* but not *C. subcristatus*, whereas 3,7,11,15-Tetramethyl-2-hexadecen-1-ol, 1,19-Eicosadiene, 17-Hydroxypregnenolone, Dodecanoic acid, Octadecedien-1-ol, Decadiene, and Stigmasterol were present in samples from *C. subcristatus* but not *C. marthae*. Examining by sex within species, the following compounds were found in samples from male but not female *C. marthae*: Neopentyl benzoate, Undecenoic acid, 1-Tridecene, Benzyl icosanoate, 1,13-Tetradecadien-3-one, Octacosyl trifluoroacetate, 1,16-Hexadecanediol, 8,11,14-Docosatrienoic acid, Androsta-4,16-dien-3-one, 9-Tetradecenoic acid, and 2-Dodecenoic acid. In contrast, Octadecanoic acid, tert-Hexadecanethiol, 2-Ethylhexyl octanoate, Campesterol, and Ergost-5-en-3-ol were present in samples from female but not male *C. marthae*. As no samples were collected for female *C. subcristatus* in 2015 sexual difference could not be examined for this species.

Supplementary Tables

Supplementary Table S 1. Compound names and their minimum (Min), 1st quartile (First_qt), median, 3rd quartile (Third_qt) and maximum (Max) relative abundance values detected in femoral pore samples collected from *C. marthae* iguanas (pink) in 2012. Values are separated for males (♂) and females (♀).

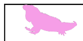	♂					♀				
Compound name	Min	First_qt	Median	Third_qt	Max	Min	First_qt	Median	Third_qt	Max
1-Heneicosanol	0.592	2.051	3.039	3.996	7.339	0.817	1.953	2.655	3.248	6.580
1-Heptadecanol	0.000	0.002	0.146	0.754	3.305	0.000	0.024	0.157	0.781	2.098
1-Hexadecanol	0.000	7.876	12.403	18.230	31.268	0.000	7.603	11.657	15.505	20.929
1-Hexadecen-3-ol, 3,5,11,15-tetramethyl-	0.000	0.000	0.000	0.000	0.791	0.000	0.000	0.000	0.000	0.123
1-Octacosanol	0.027	0.112	0.292	0.835	6.322	0.000	0.096	0.218	0.460	3.758
1-Phenyl-3-(2-phenethyl)hendecane	0.000	0.112	0.166	0.226	1.293	0.086	0.138	0.210	0.335	0.962
1-Tetradecanol	0.000	0.000	0.085	0.299	0.806	0.000	0.000	0.000	0.000	1.016
1-Tridecene	0.000	0.151	0.362	0.751	1.683	0.026	0.206	0.637	0.895	1.640
1,13-Tetradecadien-3-one	0.000	0.000	0.000	0.000	0.320	0.000	0.000	0.000	0.000	0.180
1,16-Hexadecanediol	0.000	0.000	0.101	0.249	0.687	0.000	0.000	0.098	0.204	0.976
1,19-Eicosadiene	0.000	0.000	0.000	0.000	0.230	0.000	0.000	0.000	0.000	0.129
1,37-Octatriacontadiene	0.000	0.000	0.002	0.033	7.262	0.000	0.000	0.000	0.027	0.185
10-Henicosene	0.006	5.279	11.642	18.207	32.877	0.075	5.756	10.706	15.181	43.026
10-Heptadecenoic acid	0.009	0.089	0.279	0.442	0.858	0.018	0.057	0.341	0.494	0.916
10-Methyleicosane	0.000	0.030	0.053	0.123	1.152	0.013	0.031	0.062	0.109	0.544
10-Octadecenoate	0.023	0.116	0.206	0.383	6.176	0.088	0.167	0.280	0.805	10.124
10,12-Pentacosadiynoic acid	0.000	0.000	0.000	0.000	0.112	0.000	0.000	0.000	0.000	0.024
11-Hexadecenal	0.068	0.603	0.964	1.483	5.245	0.238	0.560	0.865	1.073	6.179
11-Hexadecenoic acid	0.000	0.020	0.894	3.086	23.264	0.000	0.022	0.309	2.092	8.254
11-Octadecadien-1-ol	0.000	0.000	0.011	0.097	14.038	0.000	0.000	0.011	0.053	1.002
11-Octadecenoic acid	0.050	0.174	0.383	0.695	11.984	0.000	0.211	0.318	1.091	6.477
12-Docosanoic acid	0.000	0.131	0.207	0.379	0.861	0.000	0.063	0.211	0.314	0.574
12-Methyltetradecanoate	0.000	0.000	0.000	0.037	0.343	0.000	0.000	0.000	0.037	0.250
13-Octadecadien-1-ol	0.000	0.045	0.147	0.267	0.886	0.000	0.000	0.082	0.215	0.947
17-Hydroxypregnenolone	0.000	0.029	0.050	0.109	0.399	0.000	0.004	0.050	0.084	0.123
17-Pentatriacontene	0.000	0.000	0.054	0.231	8.684	0.000	0.000	0.043	0.144	0.241
2-(2-Butynyl)cyclohexanone	0.000	0.000	0.005	0.018	0.449	0.000	0.000	0.000	0.013	0.136
2-Dodecenoic acid	0.000	0.000	0.000	0.000	0.003	0.000	0.000	0.000	0.000	0.005
2-Ethyl-1-dodecanol	0.040	0.143	0.198	0.384	1.302	0.030	0.092	0.145	0.313	1.085
2-Ethylhexyl octanoate	0.000	0.022	0.042	0.067	0.233	0.000	0.037	0.099	0.127	0.949
2-Hepten-4-ol	0.000	0.059	0.124	0.242	1.192	0.020	0.077	0.101	0.133	0.446
2,3-Dimethyloctane	0.000	0.137	0.215	0.348	0.717	0.083	0.185	0.345	0.416	0.555
2,3,3-Trimethyloctane	0.000	0.158	0.814	1.513	7.399	0.000	1.037	1.608	3.975	9.975
2,5,5-Trimethylheptane	0.000	0.017	0.058	0.171	1.832	0.002	0.043	0.089	0.167	1.875

(continues)	♂ Min	First_qt	Median	Third_qt	Max	♀ Min	First_qt	Median	Third_qt	Max
2,6,11-Trimethyldodecane	0.035	0.366	0.673	1.124	5.612	0.137	0.813	1.290	1.827	3.847
2,7,10-Trimethyldodecane	0.000	0.161	0.329	0.570	4.345	0.100	0.207	0.503	0.808	2.617
3-Benzyloxy-1,2-diacetyl-1,2-propanediol	0.613	1.429	2.160	2.714	13.434	0.997	1.764	2.382	4.418	38.867
3-Octadecene	0.034	0.339	0.504	0.858	1.612	0.080	0.439	0.726	1.006	1.609
3,4,5,6-Tetramethyloctane	0.026	0.276	0.587	1.096	4.687	0.050	0.580	0.985	1.911	3.464
3,7,11,15-Tetramethyl-2-hexadecen-1-ol	0.000	0.000	0.000	0.000	0.035	0.000	0.000	0.000	0.000	0.031
3,8-Dimethylundecane	0.069	0.557	1.312	2.487	10.589	0.098	1.398	2.462	3.942	7.592
5-Methyl-1-heptanol	0.000	0.000	0.000	0.000	0.087	0.000	0.000	0.000	0.005	0.103
5-Methyl-5-propylnonane	0.123	0.517	0.834	2.122	5.880	0.124	1.043	1.507	2.254	3.003
7-Tetradecenal	0.000	0.000	0.003	0.025	0.077	0.000	0.000	0.000	0.007	0.093
8-Hexadecanoic acid	0.000	0.070	0.353	1.149	6.361	0.000	0.019	0.096	0.269	3.696
8,11,14-Docosatrienoic acid	0.000	0.000	0.000	0.000	0.198	0.000	0.000	0.000	0.000	0.408
9-Hexadecenal	0.000	0.048	0.104	0.509	6.491	0.000	0.047	0.077	0.128	0.697
9-Hexadecenoic acid	0.005	0.371	1.462	4.206	14.691	0.048	0.268	1.230	4.645	13.520
9-Icosene	0.000	0.360	0.547	0.830	1.533	0.273	0.539	0.706	0.854	1.603
9-Octadecene	0.000	0.088	0.172	0.335	1.458	0.020	0.087	0.113	0.267	0.895
9-Tetradecenoic acid	0.000	0.000	0.000	0.000	0.014	0.000	0.000	0.000	0.000	0.002
Alanine	0.000	0.000	0.000	0.000	0.112	0.000	0.000	0.000	0.000	0.013
Androsta-4,16-dien-3-one	0.000	0.049	0.107	0.191	1.017	0.000	0.000	0.000	0.013	0.182
Benzoquinone, 3,5-di-tert-butyl-	0.000	0.000	0.040	0.141	1.034	0.000	0.000	0.100	0.258	0.667
Benzyl icosanoate	0.000	0.000	0.111	0.207	0.739	0.000	0.000	0.000	0.224	0.721
Butenoic acid	0.000	0.070	0.107	0.163	0.585	0.000	0.016	0.054	0.137	0.259
Campesterol	0.000	0.000	0.000	0.032	3.378	0.000	0.000	0.000	0.000	0.080
Cholest-4-en-3-one	0.000	0.000	0.004	0.176	0.913	0.000	0.000	0.000	0.001	0.006
Cholesta-3,5-diene	0.000	0.000	0.099	0.209	2.211	0.000	0.110	0.190	0.243	0.375
Cholestan-3,22,26-triol	0.000	0.000	0.000	0.000	0.009	0.000	0.000	0.000	0.000	0.001
Cholestanol	0.000	0.942	1.736	2.960	7.762	0.000	0.507	1.008	2.387	3.154
Cholesterol	0.000	0.079	1.899	3.190	11.092	0.000	0.003	1.360	2.816	13.644
Crocetane	0.000	0.000	0.075	0.133	0.388	0.000	0.021	0.082	0.126	0.434
Decadiene	0.000	0.000	0.000	0.000	0.001	0.000	0.000	0.000	0.000	0.002
Decane, 5-ethyl-5-methyl-	0.000	0.029	0.045	0.086	1.210	0.000	0.023	0.048	0.083	0.270
Dimethylcapramide	0.000	0.000	0.000	0.008	0.170	0.000	0.000	0.000	0.000	0.090
Dodecane	0.000	0.142	0.246	0.502	1.603	0.017	0.364	0.486	0.819	0.996
Dodecanoic acid	0.000	0.000	0.000	0.000	0.134	0.000	0.000	0.000	0.022	0.156
Eicosane	0.000	0.443	0.722	1.004	2.115	0.000	0.052	0.390	0.928	1.941
Eicosanoic acid	0.000	1.672	3.062	5.110	13.081	0.000	0.000	2.401	3.477	7.134
Elaidyl alcohol	0.000	0.000	0.000	0.067	3.001	0.000	0.000	0.008	0.067	1.260
Ergost-5-en-3-ol	0.000	0.000	0.024	0.078	0.185	0.000	0.003	0.062	0.145	0.490
Ergost-7-en-3-ol	0.000	0.010	0.032	0.185	2.206	0.000	0.025	0.039	0.048	0.079

	♂					♀				
(continues)	Min	First_qt	Median	Third_qt	Max	Min	First_qt	Median	Third_qt	Max
Erucylamide	0.000	0.209	0.462	0.952	34.287	0.000	0.466	1.275	27.055	47.836
Ethane, 1,2-bis(9-octadecenyloxy)	0.000	0.000	0.000	0.062	13.890	0.000	0.000	0.002	0.024	0.673
Ethyl docosanoate	0.000	0.000	0.000	1.266	6.630	0.000	0.000	0.000	0.000	6.356
Heptadecyl heptadecanoate	0.000	1.000	2.282	4.597	12.082	0.000	0.227	1.554	2.952	11.602
Hexadecanal	0.000	0.084	0.150	0.347	0.792	0.038	0.124	0.269	0.345	1.059
Hexadecane	0.089	0.269	0.475	0.684	1.865	0.000	0.327	0.652	0.966	1.498
Hexadecanethiol	0.000	0.000	0.012	0.025	0.146	0.000	0.000	0.021	0.040	0.282
Hexadecenoic acid	0.000	0.269	0.788	1.339	2.422	0.000	0.047	0.624	1.228	2.559
Malonic acid	0.000	0.000	0.589	1.315	11.062	0.000	0.000	0.078	0.529	11.358
Methyl 14-methylpentadecanoate	0.000	0.000	0.046	0.947	15.789	0.000	0.000	0.086	1.100	9.054
Methyl 18-methylnonadecanoate	0.000	0.000	0.000	0.097	0.928	0.000	0.000	0.027	0.085	0.585
Methyl 3-hydroxyoctadecanoate	0.000	0.272	0.411	0.618	1.362	0.168	0.320	0.445	0.539	1.361
Methyl hexacosanoate	0.000	0.000	0.020	0.079	1.044	0.000	0.000	0.044	0.140	0.747
n-Hexatriacontane	0.000	0.000	0.099	0.181	0.435	0.000	0.019	0.086	0.171	0.534
n-Tridecanol	0.000	0.112	0.199	0.416	0.988	0.040	0.132	0.199	0.713	1.439
Neopentyl benzoate	0.000	0.000	0.000	0.020	0.253	0.000	0.000	0.000	0.016	0.068
Nonadecanoic acid	0.000	0.000	0.000	0.000	0.166	0.000	0.000	0.000	0.000	0.099
Nonahexacontanoic acid	0.000	0.026	0.048	0.079	1.163	0.000	0.038	0.061	0.090	0.164
Nonanal diethyl acetal	0.000	0.224	0.383	0.620	1.362	0.000	0.285	0.395	0.506	1.376
Octacosyl acetate	0.000	0.000	0.000	0.000	0.000	0.000	0.000	0.000	0.000	0.000
Octadecanal	0.100	0.953	1.395	3.004	13.899	0.239	0.792	1.022	1.443	8.553
Octadecanoic acid	0.000	0.016	0.074	0.203	0.825	0.000	0.000	0.000	0.054	0.213
Octadecedien-1-ol	0.000	0.000	0.000	0.000	0.014	0.000	0.000	0.000	0.005	0.021
Olealdehyde	0.034	0.199	0.405	1.154	10.774	0.085	0.212	0.318	0.619	5.213
Oleyl Alcohol	0.247	1.353	2.251	3.630	6.376	0.834	1.635	2.469	3.595	8.860
Pentadecanal-	0.010	0.029	0.046	0.082	0.921	0.019	0.038	0.078	0.392	1.051
Pentadecane	0.081	0.326	0.447	0.806	4.032	0.104	0.329	0.413	0.709	1.335
Pentadecanoic acid	0.000	0.000	0.324	0.540	1.124	0.000	0.093	0.310	0.601	1.185
Pentanedioic acid	0.000	0.099	0.188	0.622	3.035	0.000	0.109	0.347	0.617	2.486
Pregan-20-one	0.000	0.000	0.000	0.000	0.001	0.000	0.000	0.000	0.000	0.001
Pregn-4-ene-3,20-dione	0.000	0.000	0.024	0.645	2.978	0.000	0.000	0.000	0.006	0.069
Stigmasterol	0.000	0.045	0.085	0.195	0.401	0.000	0.031	0.097	0.196	0.377
Tetradecanal	0.010	0.135	0.329	0.708	4.127	0.008	0.150	0.236	0.278	1.689
Tetradecane	0.064	0.168	0.264	0.354	1.241	0.077	0.200	0.383	0.513	0.647
Tetradecene	0.000	0.000	0.000	0.000	0.217	0.000	0.000	0.000	0.000	0.002
Tetratetracontane	0.096	0.287	0.540	1.080	1.959	0.046	0.210	0.382	0.601	1.526
Tocopherol	0.000	4.551	8.958	16.093	29.282	0.552	3.762	5.707	8.315	17.884
Triacontyl acetate	0.000	0.024	0.246	1.062	6.222	0.000	0.037	0.096	0.281	3.435
Undecenoic acid	0.000	0.027	0.125	0.315	1.301	0.000	0.009	0.059	0.417	0.954

Supplementary Table S 2. Compound names and their minimum (Min), 1st quartile (First_qt), median, 3rd quartile (Third_qt) and maximum (Max) relative abundance values detected in femoral pore samples collected from *C. marthae* iguanas (pink) in 2014. Values are separated for males (♂) and females (♀).

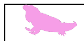	♂					♀				
Compound name	Min	First_qt	Median	Third_qt	Max	Min	First_qt	Median	Third_qt	Max
1-Heneicosanol	0.000	0.000	0.058	0.110	0.164	0.000	0.000	0.071	0.085	0.146
1-Heptadecanol	0.000	0.000	0.000	0.000	0.515	0.000	0.000	0.000	0.000	0.000
1-Hexadecanol	0.000	0.000	0.000	0.128	0.450	0.000	0.000	0.000	0.000	0.303
1-Hexadecen-3-ol, 3,5,11,15-tetramethyl-	0.000	0.000	0.000	0.000	1.641	0.000	0.000	0.000	0.000	2.133
1-Octacosanol	0.000	0.000	0.000	0.000	0.019	0.000	0.000	0.000	0.000	0.015
1-Phenyl-3-(2-phenethyl)hendecane	0.000	0.000	0.000	0.028	0.101	0.000	0.000	0.000	0.004	0.103
1-Tetradecanol	0.000	0.000	0.000	0.000	0.000	0.000	0.000	0.000	0.000	0.294
1-Tridecene	0.000	0.000	0.000	0.000	0.281	0.000	0.000	0.000	0.000	0.232
1,13-Tetradecadien-3-one	0.000	0.000	0.000	0.000	0.000	0.000	0.000	0.000	0.000	0.000
1,16-Hexadecanediol	0.000	0.000	0.000	0.000	0.069	0.000	0.000	0.000	0.000	0.396
1,19-Eicosadiene	0.000	0.000	0.000	0.000	0.009	0.000	0.000	0.000	0.000	0.015
1,37-Octatriacontadiene	0.000	0.000	0.000	0.000	0.108	0.000	0.000	0.000	0.102	0.374
10-Henicosene	0.000	0.614	1.975	2.389	3.363	0.015	0.857	1.591	1.804	2.210
10-Heptadecenoic acid	0.000	0.000	0.000	0.023	0.151	0.000	0.000	0.000	0.015	0.063
10-Methyleicosane	0.113	0.197	0.311	0.537	0.939	0.167	0.186	0.225	0.353	0.798
10-Octadecenoate	0.000	0.041	0.060	0.144	0.523	0.000	0.018	0.039	0.070	0.122
10,12-Pentacosadiynoic acid	0.000	0.000	0.000	0.000	0.000	0.000	0.000	0.000	0.000	0.000
11-Hexadecenal	0.000	0.097	1.295	2.951	14.625	0.047	0.788	3.366	6.326	13.076
11-Hexadecenoic acid	0.000	0.000	0.017	0.048	0.101	0.000	0.029	0.068	0.116	0.211
11-Octadecadien-1-ol	0.000	0.000	0.000	0.000	0.031	0.000	0.000	0.000	0.000	0.114
11-Octadecenoic acid	0.000	0.039	0.046	0.133	0.487	0.000	0.000	0.025	0.070	0.122
12-Docosanoic acid	0.000	0.000	0.000	0.000	0.000	0.000	0.000	0.000	0.000	0.009
12-Methyltetradecanoate	0.000	0.000	0.000	0.000	0.000	0.000	0.000	0.000	0.000	0.000
13-Octadecadien-1-ol	0.000	0.000	0.042	0.060	0.389	0.000	0.000	0.018	0.068	0.172
17-Hydroxypregnenolone	0.000	0.000	0.000	0.000	0.000	0.000	0.000	0.000	0.000	0.000
17-Pentatriacontene	0.011	0.033	0.184	0.729	1.004	0.000	0.012	0.037	0.081	1.598
2-(2-Butynyl)cyclohexanone	0.000	0.000	0.000	0.005	0.018	0.000	0.000	0.000	0.005	0.053
2-Dodecenoic acid	0.000	0.000	0.000	0.000	0.003	0.000	0.000	0.000	0.000	0.000
2-Ethyl-1-dodecanol	0.771	1.381	1.508	2.280	3.196	0.764	1.494	1.697	1.883	2.710
2-Ethylhexyl octanoate	0.000	0.000	0.000	0.000	0.000	0.000	0.000	0.000	0.000	0.000
2-Hepten-4-ol	0.766	1.213	1.408	1.674	2.472	0.461	1.073	1.290	1.489	1.969
2,3-Dimethyloctane	0.000	0.000	0.000	0.021	0.899	0.000	0.000	0.000	0.000	1.066
2,3,3-Trimethyloctane	0.000	0.000	0.000	0.000	0.124	0.000	0.000	0.000	0.000	0.179
2,5,5-Trimethylheptane	0.039	0.245	0.275	0.351	0.597	0.024	0.287	0.363	0.422	0.580
2,6,11-Trimethyldodecane	0.332	0.972	1.225	1.541	8.854	0.746	0.901	1.074	1.212	2.126
2,7,10-Trimethyldodecane	1.388	1.731	2.035	2.504	4.390	0.016	1.252	1.954	2.234	3.314

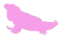	♂					♀				
(continues)	Min	First_qt	Median	Third_qt	Max	Min	First_qt	Median	Third_qt	Max
3-Benzyloxy-1,2-diacetyl-1,2-propanediol	0.004	0.049	0.124	0.191	0.252	0.008	0.104	0.143	0.170	0.399
3-Octadecene	0.000	0.000	0.000	0.000	0.000	0.000	0.000	0.000	0.000	0.000
3,4,5,6-Tetramethyloctane	0.907	0.978	1.268	1.794	2.766	0.823	1.073	1.343	1.615	2.705
3,7,11,15-Tetramethyl-2-hexadecen-1-ol	0.000	0.000	0.000	0.000	0.096	0.000	0.000	0.000	0.000	0.068
3,8-Dimethylundecane	0.648	0.936	1.149	1.548	2.554	0.530	0.959	1.302	1.495	1.818
5-Methyl-1-heptanol	0.000	0.000	0.000	0.000	0.000	0.000	0.000	0.000	0.000	0.000
5-Methyl-5-propylnonane	0.764	0.820	1.272	1.631	4.223	0.340	0.722	1.008	1.425	2.662
7-Tetradecenal	0.000	0.000	0.000	0.001	0.012	0.000	0.000	0.000	0.000	0.009
8-Hexadecanoic acid	0.000	0.000	0.000	0.005	0.026	0.000	0.000	0.004	0.018	0.036
8,11,14-Docosatrienoic acid	0.000	0.000	0.000	0.000	0.000	0.000	0.000	0.000	0.000	0.082
9-Hexadecenal	0.000	0.023	0.081	0.103	0.112	0.018	0.054	0.084	0.122	0.304
9-Hexadecenoic acid	0.040	0.236	0.651	1.031	1.589	0.018	0.047	0.616	0.715	1.171
9-Icosene	0.000	0.000	0.000	0.000	0.000	0.000	0.000	0.000	0.000	0.930
9-Octadecene	0.702	1.282	2.508	6.256	16.179	1.135	1.441	1.484	2.022	7.909
9-Tetradecenoic acid	0.000	0.000	0.000	0.000	0.016	0.000	0.000	0.000	0.000	0.000
Alanine	0.000	0.000	0.000	0.000	0.000	0.000	0.000	0.000	0.000	0.000
Androsta-4,16-dien-3-one	0.000	0.000	0.000	0.000	0.000	0.000	0.000	0.000	0.000	0.010
Benzoquinone, 3,5-di-tert-butyl-	0.000	0.000	0.000	0.000	0.000	0.000	0.000	0.000	0.000	0.000
Benzyl icosanoate	0.000	0.000	0.000	0.000	0.000	0.000	0.000	0.000	0.000	0.000
Butenoic acid	0.211	0.305	0.344	0.391	0.689	0.216	0.339	0.446	0.635	1.171
Campesterol	0.000	0.000	0.000	0.000	0.000	0.000	0.000	0.000	0.000	0.000
Cholest-4-en-3-one	0.000	0.000	0.000	0.004	0.010	0.000	0.000	0.000	0.002	0.024
Cholesta-3,5-diene	0.000	0.000	1.610	2.816	4.718	0.000	0.000	0.000	3.405	7.476
Cholestan-3,22,26-triol	0.000	0.000	0.000	0.000	0.000	0.000	0.000	0.000	0.000	0.000
Cholestanol	0.000	0.000	0.000	0.000	0.000	0.000	0.000	0.000	0.000	0.000
Cholesterol	0.000	0.000	0.000	0.000	0.000	0.000	0.000	0.000	0.000	0.013
Crocetane	0.290	0.444	0.524	0.889	1.376	0.375	0.506	0.717	1.025	1.455
Decadiene	0.000	0.000	0.000	0.000	0.001	0.000	0.000	0.000	0.000	0.002
Decane, 5-ethyl-5-methyl-	0.043	0.099	0.149	0.162	0.292	0.084	0.105	0.136	0.225	0.938
Dimethylcapramide	0.000	0.000	0.000	0.000	0.000	0.000	0.000	0.000	0.000	0.000
Dodecane	0.379	0.725	0.866	1.016	1.859	0.274	0.645	0.866	1.010	1.801
Dodecanoic acid	0.000	0.000	0.000	0.000	0.000	0.000	0.000	0.000	0.000	0.000
Eicosane	1.311	3.101	4.858	7.312	10.187	2.508	4.074	5.747	7.095	11.286
Eicosanoic acid	0.000	0.000	0.000	0.000	0.000	0.000	0.000	0.000	0.000	0.000
Elaidyl alcohol	0.000	0.000	0.000	0.000	0.000	0.000	0.000	0.000	0.000	0.017
Ergost-5-en-3-ol	0.000	0.000	0.000	0.000	0.000	0.000	0.000	0.000	0.000	0.008
Ergost-7-en-3-ol	0.000	0.000	0.000	0.000	0.002	0.000	0.000	0.000	0.005	0.029
Erucylamide	0.000	0.000	0.000	0.000	0.001	0.000	0.000	0.000	0.000	0.000
Ethane, 1,2-bis(9-octadecenyloxy)	0.000	0.000	0.000	0.000	0.060	0.000	0.000	0.010	0.039	0.114

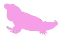	♂					♀				
(continues)	Min	First_qt	Median	Third_qt	Max	Min	First_qt	Median	Third_qt	Max
Ethyl docosanoate	0.000	0.000	0.000	0.000	0.000	0.000	0.000	0.000	0.967	7.207
Heptadecyl heptadecanoate	0.000	0.332	1.214	3.743	5.932	0.000	0.199	0.642	1.425	5.545
Hexadecanal	0.018	0.404	0.867	6.420	14.704	0.386	0.574	1.372	2.592	8.784
Hexadecane	0.010	1.323	2.035	2.223	4.390	0.023	0.051	1.286	1.833	3.314
Hexadecanethiol	0.000	0.019	0.094	0.109	0.287	0.000	0.060	0.077	0.164	0.335
Hexadecenoic acid	0.000	1.013	7.809	10.352	19.048	4.686	7.004	7.780	10.733	20.530
Malonic acid	0.000	0.000	0.000	0.000	0.000	0.000	0.000	0.000	0.000	0.000
Methyl 14-methylpentadecanoate	0.000	0.000	0.000	0.000	0.000	0.000	0.000	0.000	0.000	0.000
Methyl 18-methylnonadecanoate	0.000	0.000	0.000	0.000	0.000	0.000	0.000	0.000	0.012	0.025
Methyl 3-hydroxyoctadecanoate	0.000	0.000	0.000	0.000	0.188	0.000	0.000	0.000	0.000	0.221
Methyl hexacosanoate	0.000	0.000	0.000	0.000	0.037	0.000	0.000	0.000	0.000	0.000
n-Hexatriacontane	0.440	0.599	1.137	1.504	1.769	0.518	0.882	1.030	1.333	1.571
n-Tridecanol	0.149	5.813	13.581	20.247	27.419	0.338	0.448	13.370	18.066	23.892
Neopentyl benzoate	0.000	0.000	0.000	0.000	0.000	0.000	0.000	0.000	0.000	0.000
Nonadecanoic acid	0.000	0.000	0.000	0.000	0.000	0.000	0.000	0.000	0.000	0.000
Nonahexacontanoic acid	0.164	0.385	0.463	0.780	1.819	0.188	0.353	0.573	1.107	3.116
Nonanal diethyl acetal	0.000	0.000	0.000	0.000	0.068	0.000	0.000	0.000	0.000	0.052
Octacosyl acetate	0.015	0.131	0.160	0.236	0.276	0.094	0.134	0.170	0.218	0.456
Octadecanal	0.000	6.714	7.902	10.332	13.176	0.000	3.629	8.869	10.010	12.606
Octadecanoic acid	0.000	0.000	0.000	0.000	0.000	0.000	0.000	0.000	0.000	0.003
Octadecedien-1-ol	0.000	0.000	0.000	0.000	0.004	0.000	0.000	0.000	0.000	0.000
Olealdehyde	0.387	10.550	12.520	15.556	24.111	8.967	13.398	17.783	24.271	29.167
Oleyl Alcohol	0.000	0.000	0.207	0.816	2.018	0.000	0.062	0.121	1.198	3.067
Pentadecanal-	0.000	0.000	0.000	0.000	1.307	0.000	0.000	0.000	0.000	0.000
Pentadecane	0.147	0.209	0.725	1.072	3.683	0.131	0.236	1.178	1.607	3.023
Pentadecanoic acid	0.000	0.000	0.000	0.000	0.000	0.000	0.000	0.000	0.000	0.000
Pentanedioic acid	0.000	0.000	0.000	0.000	0.000	0.000	0.000	0.000	0.000	0.000
Pregan-20-one	0.000	0.000	0.000	0.000	0.001	0.000	0.000	0.000	0.000	0.000
Pregn-4-ene-3,20-dione	0.000	0.000	0.376	0.612	1.163	0.000	0.236	0.491	1.422	3.402
Stigmasterol	0.020	0.042	0.073	0.107	0.229	0.026	0.108	0.249	0.319	0.761
Tetradecanal	0.000	0.000	8.509	12.402	15.422	0.000	0.000	0.000	4.523	17.194
Tetradecane	0.758	1.256	1.598	2.357	3.394	0.711	1.272	1.509	1.725	2.529
Tetradecene	0.000	0.011	0.026	0.044	0.074	0.019	0.033	0.041	0.056	0.128
Tetratetracontane	0.134	0.343	0.560	0.636	1.026	0.014	0.248	0.431	0.773	5.112
Tocopherol	0.000	0.000	0.000	0.000	0.000	0.000	0.000	0.000	0.000	0.000
Triacontyl acetate	0.000	0.000	0.000	0.022	0.033	0.000	0.000	0.000	0.009	0.036
Undecenoic acid	0.000	0.000	0.000	0.000	0.000	0.000	0.000	0.000	0.000	0.000

Supplementary Table S 3. Compound names and their minimum (Min), 1st quartile (First_qt), median, 3rd quartile (Third_qt) and maximum (Max) relative abundance values detected in femoral pore samples collected from *C. marthae* iguanas (pink) in 2015. Values are separated for males (♂) and females (♀).

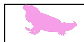	♂					♀				
Compound name	Min	First_qt	Median	Third_qt	Max	Min	First_qt	Median	Third_qt	Max
1-Heneicosanol	0.067	0.188	0.193	0.389	0.987	0.000	0.059	0.118	0.207	0.295
1-Heptadecanol	0.000	0.000	0.000	0.305	3.078	0.471	1.708	2.944	3.095	3.247
1-Hexadecanol	0.000	0.000	0.265	0.313	0.663	0.000	0.140	0.281	0.327	0.373
1-Hexadecen-3-ol, 3,5,11,15-tetramethyl-	0.000	0.000	0.000	0.574	0.652	0.000	0.000	0.000	0.128	0.256
1-Octacosanol	0.009	0.026	0.029	0.059	0.099	0.003	0.010	0.017	0.074	0.130
1-Phenyl-3-(2-phenethyl)hendecane	0.000	0.000	0.000	0.000	0.000	0.000	0.000	0.000	0.000	0.000
1-Tetradecanol	0.274	0.306	0.369	0.371	0.593	0.271	0.332	0.393	0.455	0.517
1-Tridecene	0.000	0.000	0.000	0.076	0.107	0.000	0.000	0.000	0.000	0.000
1,13-Tetradecadien-3-one	0.000	0.000	0.000	0.002	0.153	0.000	0.000	0.000	0.000	0.000
1,16-Hexadecanediol	0.000	0.000	0.000	0.176	0.495	0.000	0.000	0.000	0.000	0.000
1,19-Eicosadiene	0.000	0.000	0.000	0.000	0.000	0.000	0.000	0.000	0.000	0.000
1,37-Octatriacontadiene	0.067	0.223	0.269	0.319	1.977	0.100	0.936	1.772	2.360	2.949
10-Henicosene	0.000	0.000	0.000	0.663	0.736	0.000	0.000	0.000	0.030	0.061
10-Heptadecenoic acid	6.819	7.052	7.661	11.521	12.986	6.026	6.380	6.735	6.974	7.214
10-Methyleicosane	0.103	0.104	0.107	0.146	0.205	0.104	0.118	0.132	1.950	3.769
10-Octadecenoate	0.399	2.699	3.472	4.997	8.336	0.186	2.184	4.181	5.107	6.034
10,12-Pentacosadiynoic acid	0.000	0.000	0.000	0.000	0.000	0.000	0.000	0.000	0.000	0.000
11-Hexadecenal	0.288	0.294	0.464	0.630	0.917	0.060	0.067	0.073	0.814	1.556
11-Hexadecenoic acid	0.120	0.234	0.234	0.273	0.827	0.038	0.118	0.198	1.206	2.213
11-Octadecadien-1-ol	0.000	0.000	0.000	0.056	0.105	0.000	0.000	0.000	0.032	0.064
11-Octadecenoic acid	0.399	0.400	2.699	3.472	4.997	0.186	2.184	4.181	5.107	6.034
12-Docosanoic acid	0.011	0.042	0.186	0.299	0.378	0.002	0.033	0.064	0.198	0.333
12-Methyltetradecanoate	0.000	0.000	2.026	2.284	2.776	0.000	1.881	3.761	4.284	4.808
13-Octadecadien-1-ol	0.000	0.000	0.000	0.000	0.294	0.000	0.124	0.248	0.250	0.253
17-Hydroxypregnenolone	0.000	0.000	0.000	0.000	0.000	0.000	0.000	0.000	0.000	0.000
17-Pentatriacontene	0.000	0.000	0.000	0.138	0.149	0.003	0.020	0.036	0.068	0.100
2-(2-Butynyl)cyclohexanone	0.000	0.000	0.000	0.004	0.007	0.000	0.000	0.000	0.010	0.021
2-Dodecenoic acid	0.000	0.008	0.012	0.018	0.031	0.000	0.000	0.000	0.000	0.000
2-Ethyl-1-dodecanol	0.000	0.000	0.417	0.700	2.429	0.580	0.615	0.650	0.939	1.227
2-Ethylhexyl octanoate	0.000	0.000	0.000	0.000	0.000	0.000	0.000	0.000	0.126	0.253
2-Hepten-4-ol	0.095	0.095	0.105	0.122	0.139	0.239	0.491	0.743	1.843	2.942
2,3-Dimethyloctane	0.241	0.287	0.303	0.350	0.450	0.201	0.274	0.346	0.375	0.405
2,3,3-Trimethyloctane	0.000	0.000	0.199	0.226	0.280	0.079	0.207	0.336	0.410	0.484
2,5,5-Trimethylheptane	0.065	0.077	0.093	0.156	0.183	0.050	0.100	0.151	0.154	0.158
2,6,11-Trimethyldodecane	0.000	0.189	0.253	0.316	2.242	0.387	0.481	0.576	0.651	0.726
2,7,10-Trimethyldodecane	0.003	0.027	0.033	0.227	0.481	0.169	0.291	0.412	0.413	0.414

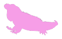

(continues)	♂ Min	First_qt	Median	Third_qt	Max	♀ Min	First_qt	Median	Third_qt	Max
3-Benzyloxy-1,2-diacetyl-1,2-propanediol	0.061	0.082	0.087	0.198	0.237	0.011	0.031	0.050	0.193	0.336
3-Octadecene	0.375	0.421	0.458	1.344	2.398	0.255	0.591	0.927	1.007	1.088
3,4,5,6-Tetramethyloctane	0.173	0.208	0.290	0.421	0.807	0.245	0.376	0.507	0.531	0.556
3,7,11,15-Tetramethyl-2-hexadecen-1-ol	0.000	0.000	0.000	0.000	0.000	0.000	0.000	0.000	0.000	0.000
3,8-Dimethylundecane	0.157	0.157	0.229	0.264	0.368	0.240	0.339	0.437	0.473	0.509
5-Methyl-1-heptanol	0.000	0.000	0.000	0.000	0.000	0.000	0.000	0.000	0.000	0.000
5-Methyl-5-propylnonane	0.245	0.591	0.746	0.864	1.081	0.776	0.903	1.029	1.137	1.246
7-Tetradecenal	0.000	0.000	0.000	0.008	0.017	0.000	0.000	0.000	0.006	0.012
8-Hexadecanoic acid	0.021	0.026	0.029	0.057	0.062	0.002	0.033	0.063	0.092	0.122
8,11,14-Docosatrienoic acid	0.000	0.000	0.000	0.658	1.434	0.000	0.000	0.000	0.000	0.000
9-Hexadecenal	0.130	0.194	0.196	0.217	0.239	0.253	0.268	0.284	0.530	0.776
9-Hexadecenoic acid	6.311	7.555	8.489	9.939	11.728	2.913	5.355	7.798	8.067	8.337
9-Icosene	1.431	2.730	4.404	10.460	11.207	1.028	1.683	2.338	2.383	2.429
9-Octadecene	0.276	2.194	2.389	2.587	2.994	0.213	0.340	0.466	0.500	0.534
9-Tetradecenoic acid	0.000	0.000	0.000	0.000	0.001	0.000	0.000	0.000	0.000	0.000
Alanine	0.000	0.000	0.000	0.000	0.000	0.000	0.000	0.000	0.000	0.000
Androsta-4,16-dien-3-one	0.000	0.000	0.000	0.004	0.021	0.000	0.000	0.000	0.000	0.000
Benzoquinone, 3,5-di-tert-butyl-	0.000	0.000	0.000	0.000	0.000	0.000	0.000	0.000	0.000	0.000
Benzyl icosanoate	0.000	0.000	0.026	0.063	0.521	0.000	0.000	0.000	0.000	0.000
Butenoic acid	0.148	0.177	0.211	0.214	0.363	0.164	0.228	0.292	0.359	0.427
Campesterol	0.000	0.000	0.000	0.000	0.000	0.000	0.076	0.152	0.171	0.189
Cholest-4-en-3-one	0.000	0.000	0.000	0.000	0.013	0.000	0.000	0.000	0.001	0.002
Cholesta-3,5-diene	0.520	0.557	0.648	0.914	1.801	0.000	0.480	0.960	0.964	0.969
Cholestan-3,22,26-triol	0.000	0.000	0.000	0.000	0.000	0.000	0.000	0.000	0.000	0.000
Cholestanol	0.000	0.000	0.000	0.000	0.000	0.000	0.000	0.000	0.000	0.000
Cholesterol	0.000	0.000	0.000	0.000	0.000	0.000	0.000	0.000	0.000	0.000
Crocetane	0.213	0.254	0.309	0.312	0.371	0.194	0.292	0.390	0.413	0.437
Decadiene	0.000	0.000	0.000	0.000	0.000	0.000	0.000	0.000	0.000	0.000
Decane, 5-ethyl-5-methyl-	0.144	0.171	0.200	0.204	0.224	0.128	0.172	0.216	0.229	0.242
Dimethylcapramide	0.301	0.326	0.506	0.862	1.050	0.227	0.248	0.269	0.517	0.766
Dodecane	0.228	0.260	0.392	0.575	0.732	0.080	0.106	0.133	0.250	0.367
Dodecanoic acid	0.000	0.000	0.000	0.000	0.000	0.000	0.000	0.000	0.000	0.000
Eicosane	0.401	0.417	0.437	0.442	0.455	0.178	0.377	0.576	0.857	1.139
Eicosanoic acid	0.000	0.000	0.000	0.000	0.000	0.000	0.000	0.000	0.000	0.000
Elaidyl alcohol	0.000	0.000	0.000	0.000	0.499	0.000	0.000	0.000	0.628	1.255
Ergost-5-en-3-ol	0.000	0.000	0.000	0.000	0.000	0.000	0.014	0.027	0.043	0.059
Ergost-7-en-3-ol	0.191	0.338	0.584	0.615	0.796	0.484	0.500	0.516	0.639	0.763
Erucylamide	0.000	0.000	0.000	0.000	0.000	0.000	0.000	0.000	0.000	0.000
Ethane, 1,2-bis(9-octadecenyloxy)	1.864	2.082	2.347	2.389	3.135	0.246	1.290	2.334	2.905	3.477

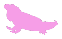	♂					♀				
(continues)	Min	First_qt	Median	Third_qt	Max	Min	First_qt	Median	Third_qt	Max
Ethyl docosanoate	0.000	0.000	0.000	0.000	0.000	0.000	0.000	0.000	0.000	0.000
Heptadecyl heptadecanoate	0.305	0.308	0.308	0.327	0.614	0.286	1.125	1.964	1.976	1.990
Hexadecanal	0.000	0.000	0.033	0.157	0.872	0.000	0.138	0.276	0.356	0.436
Hexadecane	0.019	0.027	0.308	0.590	0.781	0.306	0.414	0.523	0.584	0.646
Hexadecanethiol	0.000	0.000	0.000	0.000	0.000	0.141	0.183	0.226	0.290	0.354
Hexadecenoic acid	3.999	9.295	10.977	12.049	13.753	6.032	7.828	9.625	9.767	9.909
Malonic acid	0.000	0.000	0.000	0.000	0.000	0.000	0.000	0.000	0.000	0.000
Methyl 14-methylpentadecanoate	0.121	0.583	0.630	1.488	3.047	1.352	1.535	1.718	1.723	1.728
Methyl 18-methylnonadecanoate	10.060	10.990	12.610	12.990	13.740	13.620	13.740	13.860	15.550	17.250
Methyl 3-hydroxyoctadecanoate	0.000	0.000	0.000	0.000	0.000	0.000	0.000	0.000	0.000	0.000
Methyl hexacosanoate	0.473	5.380	8.574	9.995	10.855	6.065	9.436	12.807	13.455	14.104
n-Hexatriacontane	0.000	0.196	0.275	0.355	0.371	0.215	0.322	0.430	0.433	0.437
n-Tridecanol	4.263	4.438	10.308	13.849	15.903	1.061	1.641	2.222	2.314	2.406
Neopentyl benzoate	0.000	0.000	0.000	0.000	0.006	0.000	0.000	0.000	0.000	0.000
Nonadecanoic acid	0.000	0.000	0.000	0.000	0.000	0.000	0.000	0.000	0.000	0.000
Nonahexacontanoic acid	0.066	0.151	0.174	0.214	0.263	0.292	0.297	0.303	1.382	2.461
Nonanal diethyl acetal	0.000	0.000	0.000	0.000	0.000	0.000	0.000	0.000	0.000	0.000
Octacosyl acetate	0.000	0.000	0.000	0.000	0.063	0.000	0.000	0.000	0.000	0.000
Octadecanal	0.000	0.000	0.000	0.956	3.355	0.780	1.388	1.995	2.208	2.421
Octadecanoic acid	0.000	0.000	0.000	0.000	0.000	0.000	0.000	0.000	0.024	0.048
Octadecedien-1-ol	0.000	0.000	0.000	0.000	0.000	0.000	0.000	0.000	0.000	0.000
Olealdehyde	0.294	0.299	0.398	0.535	2.289	0.045	0.054	0.064	0.175	0.287
Oleyl Alcohol	0.000	0.000	0.000	0.000	0.249	0.102	0.258	0.413	0.521	0.629
Pentadecanal-	0.000	0.000	0.000	0.537	0.638	0.000	0.000	0.000	0.137	0.275
Pentadecane	0.000	0.016	0.031	0.080	0.123	0.000	0.015	0.030	0.207	0.383
Pentadecanoic acid	0.000	0.000	0.000	0.000	0.000	0.000	0.000	0.000	0.000	0.000
Pentanedioic acid	0.000	0.000	0.000	0.000	0.000	0.000	0.000	0.000	0.000	0.000
Pregan-20-one	0.000	0.000	0.000	0.000	0.000	0.000	0.000	0.000	0.000	0.000
Pregn-4-ene-3,20-dione	2.567	4.335	4.676	4.795	4.871	1.679	2.639	3.599	3.924	4.249
Stigmasterol	0.000	0.000	0.000	0.000	0.000	0.000	0.000	0.000	0.000	0.000
Sulfurous acid	0.000	0.000	0.000	0.000	0.324	0.000	0.100	0.201	0.278	0.355
Tetradecanal	0.081	0.154	0.164	0.170	0.176	0.000	0.000	0.000	0.098	0.196
Tetradecane	0.386	0.437	0.497	0.593	0.737	0.287	0.499	0.711	0.767	0.824
Tetradecene	0.016	0.016	0.016	0.018	0.019	0.000	0.011	0.021	0.026	0.030
Tetratetracontane	0.113	0.174	0.200	0.208	0.352	0.093	0.126	0.159	0.567	0.975
Tocopherol	0.000	0.000	0.000	0.000	0.000	0.000	0.000	0.000	0.000	0.000
Triacontyl acetate	0.029	0.046	0.050	0.057	0.064	0.024	0.044	0.063	0.364	0.665
Undecenoic acid	0.000	0.000	0.000	0.000	0.043	0.000	0.000	0.000	0.000	0.000

Supplementary Table S 4. Compound names and their minimum (Min), 1st quartile (First_qt), median, 3rd quartile (Third_qt) and maximum (Max) relative abundance values detected in femoral pore samples collected from *C. subcristatus* iguanas (yellow) in 2012. Values are separated for males (♂) and females (♀).

	♂					♀				
Compound name	Min	First_qt	Median	Third_qt	Max	Min	First_qt	Median	Third_qt	Max
1-Heneicosanol	0.000	0.123	0.251	0.440	1.822	0.000	0.175	0.418	0.886	2.471
1-Heptadecanol	0.000	0.000	0.025	0.143	1.733	0.000	0.010	0.384	1.080	3.371
1-Hexadecanol	0.000	0.717	1.154	2.158	5.051	0.765	1.941	3.170	6.056	9.020
1-Hexadecen-3-ol, 3,5,11,15-tetramethyl-	0.000	0.000	0.000	0.025	0.468	0.000	0.000	0.000	0.000	0.389
1-Octacosanol	0.000	0.135	0.396	1.365	5.210	0.000	0.065	0.379	1.288	6.717
1-Phenyl-3-(2-phenethyl)hendecane	0.089	0.165	0.235	0.410	0.998	0.000	0.155	0.223	0.355	0.566
1-Tetradecanol	0.000	0.000	0.000	0.000	0.218	0.000	0.000	0.000	0.069	0.260
1-Tridecene	0.059	0.246	0.806	1.157	2.282	0.000	0.114	0.361	0.695	1.729
1,13-Tetradecadien-3-one	0.000	0.000	0.000	0.000	0.635	0.000	0.000	0.000	0.000	0.075
1,16-Hexadecanediol	0.000	0.000	0.000	0.000	0.025	0.000	0.000	0.000	0.000	0.178
1,19-Eicosadiene	0.000	0.000	0.000	0.000	0.274	0.000	0.000	0.000	0.000	0.028
1,37-Octatriacontadiene	0.000	0.000	0.020	0.369	3.019	0.000	0.000	0.000	0.041	0.137
10-Henicosene	0.000	0.275	0.777	1.336	5.045	0.056	1.822	2.962	5.191	10.691
10-Heptadecenoic acid	0.000	0.041	0.081	0.162	0.619	0.014	0.064	0.135	0.280	0.943
10-Methyleicosane	0.000	0.022	0.051	0.163	0.631	0.000	0.025	0.049	0.226	1.487
10-Octadecenoate	0.004	0.201	0.298	0.818	11.099	0.000	0.217	0.360	0.806	4.319
10,12-Pentacosadiynoic acid	0.000	0.000	0.000	0.000	0.201	0.000	0.000	0.000	0.000	0.029
11-Hexadecenal	0.052	0.334	0.601	1.317	5.545	0.136	0.435	0.577	1.067	4.087
11-Hexadecenoic acid	0.000	0.005	0.421	4.538	22.940	0.000	0.010	0.896	5.208	19.771
11-Octadecadien-1-ol	0.000	0.009	0.056	0.259	3.003	0.000	0.000	0.018	0.087	0.826
11-Octadecenoic acid	0.055	0.235	0.639	1.620	7.121	0.072	0.252	0.471	1.040	9.911
12-Docosanoic acid	0.000	0.019	0.050	0.126	0.396	0.000	0.040	0.085	0.153	0.774
12-Methyltetradecanoate	0.000	0.000	0.000	0.000	0.128	0.000	0.000	0.000	0.000	0.222
13-Octadecadien-1-ol	0.000	0.000	0.000	0.041	0.189	0.000	0.000	0.000	0.021	0.293
17-Hydroxypregnenolone	0.000	0.000	0.157	0.402	0.816	0.036	0.133	0.192	0.327	0.901
17-Pentatriacontene	0.000	0.000	0.030	0.541	3.735	0.000	0.000	0.016	0.102	0.499
2-(2-Butynyl)cyclohexanone	0.000	0.000	0.008	0.026	0.456	0.000	0.000	0.008	0.017	0.139
2-Dodecenoic acid	0.000	0.000	0.000	0.000	0.004	0.000	0.000	0.000	0.000	0.003
2-Ethyl-1-dodecanol	0.037	0.168	0.249	0.370	1.617	0.041	0.130	0.271	0.388	1.203
2-Ethylhexyl octanoate	0.000	0.023	0.048	0.093	0.693	0.000	0.048	0.086	0.153	1.109
2-Hepten-4-ol	0.000	0.117	0.225	0.445	1.605	0.000	0.050	0.100	0.256	1.495
2,3-Dimethyloctane	0.014	0.222	0.319	0.500	1.028	0.035	0.151	0.223	0.362	0.867
2,3,3-Trimethyloctane	0.000	0.405	1.296	2.647	10.459	0.000	0.239	0.637	2.336	11.421
2,5,5-Trimethylheptane	0.000	0.025	0.115	0.203	1.295	0.000	0.039	0.098	0.152	5.648
2,6,11-Trimethyldodecane	0.069	0.551	1.183	1.891	7.224	0.142	0.414	0.632	1.509	6.101
2,7,10-Trimethyldodecane	0.068	0.273	0.608	1.313	16.723	0.000	0.166	0.272	0.630	3.010

	♂					♀				
(continues)	Min	First_qt	Median	Third_qt	Max	Min	First_qt	Median	Third_qt	Max
3-Benzyloxy-1,2-diacetyl-1,2-propanediol	1.121	1.971	3.062	5.150	8.562	0.786	1.937	2.470	3.996	7.120
3-Octadecene	0.113	0.461	0.912	1.446	2.490	0.025	0.320	0.709	0.965	1.683
3,4,5,6-Tetramethyloctane	0.000	0.341	1.000	1.838	6.956	0.004	0.158	0.419	1.280	4.848
3,7,11,15-Tetramethyl-2-hexadecen-1-ol	0.000	0.000	0.000	0.000	0.028	0.000	0.000	0.000	0.000	0.008
3,8-Dimethylundecane	0.000	0.805	2.178	3.886	17.407	0.114	0.507	1.070	2.671	11.305
5-Methyl-1-heptanol	0.000	0.000	0.000	0.000	0.151	0.000	0.000	0.000	0.000	0.049
5-Methyl-5-propylnonane	0.160	0.533	1.007	2.246	8.415	0.164	0.444	0.811	1.783	6.473
7-Tetradecenal	0.000	0.000	0.002	0.022	0.302	0.000	0.000	0.000	0.018	0.101
8-Hexadecanoic acid	0.000	0.040	0.207	1.208	12.365	0.000	0.071	0.245	1.643	6.279
8,11,14-Docosatrienoic acid	0.000	0.000	0.000	0.000	0.319	0.000	0.000	0.000	0.000	0.000
9-Hexadecenal	0.000	0.035	0.276	1.502	2.748	0.000	0.066	0.187	0.380	2.586
9-Hexadecenoic acid	0.000	0.207	1.236	6.424	18.629	0.000	0.137	0.403	2.585	22.905
9-Icosene	0.000	0.462	0.860	1.322	2.208	0.126	0.406	0.646	0.989	2.091
9-Octadecene	0.000	0.111	0.172	0.297	2.328	0.017	0.069	0.157	0.323	2.411
9-Tetradecenoic acid	0.000	0.000	0.000	0.000	0.014	0.000	0.000	0.000	0.000	0.012
Alanine	0.000	0.000	0.000	0.000	0.028	0.000	0.000	0.000	0.000	0.024
Androsta-4,16-dien-3-one	0.000	0.000	0.000	0.010	0.041	0.000	0.000	0.000	0.012	0.866
Benzoquinone, 3,5-di-tert-butyl-	0.000	0.000	0.000	0.163	0.402	0.000	0.000	0.050	0.195	3.037
Benzyl icosanoate	0.000	0.000	0.232	0.345	0.792	0.000	0.000	0.152	0.255	0.650
Butenoic acid	0.000	0.000	0.000	0.008	0.193	0.000	0.000	0.000	0.009	0.481
Campesterol	0.000	0.000	0.000	0.000	1.741	0.000	0.000	0.000	0.000	0.436
Cholest-4-en-3-one	0.000	0.000	0.000	0.000	0.178	0.000	0.000	0.000	0.001	0.167
Cholesta-3,5-diene	0.000	0.084	0.218	0.348	3.445	0.000	0.063	0.137	0.256	0.731
Cholestan-3,22,26-triol	0.000	0.000	0.000	0.000	0.001	0.000	0.000	0.000	0.000	0.004
Cholestanol	0.000	0.000	0.000	0.472	1.138	0.000	0.000	0.000	0.115	0.656
Cholesterol	0.000	0.198	1.581	5.417	31.631	0.000	0.000	0.000	0.393	14.948
Crocetane	0.000	0.000	0.040	0.207	1.162	0.000	0.000	0.000	0.191	0.539
Decadiene	0.000	0.000	0.000	0.000	0.011	0.000	0.000	0.000	0.000	0.002
Decane, 5-ethyl-5-methyl-	0.000	0.031	0.061	0.144	0.558	0.000	0.000	0.030	0.072	0.227
Dimethylcapramide	0.000	0.000	0.000	0.008	0.441	0.000	0.000	0.000	0.000	0.179
Dodecane	0.000	0.116	0.294	0.580	3.125	0.086	0.137	0.333	0.676	2.415
Dodecanoic acid	0.000	0.000	0.000	0.000	0.035	0.000	0.000	0.000	0.000	0.094
Eicosane	0.000	0.433	0.671	1.153	2.244	0.000	0.115	0.452	0.784	2.341
Eicosanoic acid	0.000	0.540	8.272	12.953	22.700	0.000	0.000	3.385	7.711	21.814
Elaidyl alcohol	0.000	0.000	0.000	0.000	1.529	0.000	0.000	0.000	0.114	0.643
Ergost-5-en-3-ol	0.000	0.000	0.029	0.091	0.376	0.000	0.000	0.036	0.094	0.221
Ergost-7-en-3-ol	0.000	0.000	0.040	0.076	0.922	0.000	0.000	0.019	0.033	1.154
Erucylamide	0.000	0.215	0.619	0.887	3.374	0.155	0.643	2.863	20.194	47.700
Ethane, 1,2-bis(9-octadecenyloxy)	0.000	0.000	0.000	0.026	2.915	0.000	0.000	0.009	0.027	0.802

	♂					♀				
(continues)	Min	First_qt	Median	Third_qt	Max	Min	First_qt	Median	Third_qt	Max
Ethyl docosanoate	0.000	0.000	0.000	0.000	5.212	0.000	0.000	0.000	0.020	5.319
Heptadecyl heptadecanoate	0.000	1.573	3.145	5.984	18.834	0.000	0.569	3.811	6.663	12.900
Hexadecanal	0.000	0.106	0.232	0.448	1.410	0.000	0.066	0.113	0.284	1.826
Hexadecane	0.145	0.414	0.576	0.869	2.777	0.113	0.301	0.421	0.739	2.690
Hexadecanethiol	0.000	0.000	0.011	0.041	0.326	0.000	0.000	0.000	0.022	0.307
Hexadecenoic acid	0.000	0.000	0.016	0.061	0.177	0.000	0.006	0.189	0.450	0.865
Malonic acid	0.000	0.000	0.000	1.658	60.264	0.000	0.026	0.449	1.537	7.987
Methyl 14-methylpentadecanoate	0.000	0.000	0.788	2.290	7.512	0.000	0.000	0.474	1.301	8.604
Methyl 18-methylnonadecanoate	0.000	0.000	0.000	0.000	0.576	0.000	0.000	0.000	0.000	0.497
Methyl 3-hydroxyoctadecanoate	0.000	0.458	0.701	0.918	2.241	0.000	0.648	1.098	2.575	4.113
Methyl hexacosanoate	0.000	0.000	0.000	0.000	0.535	0.000	0.000	0.000	0.002	0.654
n-Hexatriacontane	0.000	0.000	0.040	0.214	0.740	0.000	0.000	0.024	0.109	0.415
n-Tridecanol	0.000	0.420	0.826	1.312	2.208	0.020	0.183	0.626	0.879	2.078
Neopentyl benzoate	0.000	0.000	0.000	0.000	0.428	0.000	0.000	0.000	0.000	0.091
Nonadecanoic acid	0.000	0.000	0.000	0.000	0.333	0.000	0.000	0.000	0.000	0.211
Nonahexacontanoic acid	0.000	0.015	0.080	0.177	1.105	0.000	0.000	0.036	0.078	1.561
Nonanal diethyl acetal	0.000	0.337	0.479	0.690	1.613	0.000	0.434	0.720	1.064	5.101
Octacosyl acetate	0.000	0.000	0.000	0.000	0.000	0.000	0.000	0.000	0.000	0.000
Octadecanal	0.355	1.437	2.405	4.436	9.537	0.007	0.524	0.983	1.683	11.779
Octadecanoic acid	0.000	0.000	0.000	0.000	0.044	0.000	0.000	0.000	0.000	0.825
Octadecedien-1-ol	0.000	0.000	0.000	0.000	0.017	0.000	0.000	0.000	0.000	0.001
Olealdehyde	0.095	0.515	0.972	2.371	41.816	0.028	0.302	1.538	9.285	47.561
Oleyl Alcohol	0.000	0.000	0.062	0.317	3.112	0.000	0.000	0.210	0.497	2.836
Pentadecanal-	0.020	0.041	0.080	0.125	1.068	0.011	0.029	0.049	0.330	1.193
Pentadecane	0.157	0.516	0.803	1.166	2.783	0.037	0.256	0.358	0.790	1.720
Pentadecanoic acid	0.000	0.000	0.615	1.253	3.153	0.000	0.365	0.678	0.981	3.189
Pentanedioic acid	0.000	0.121	0.279	0.697	3.974	0.000	0.114	0.271	0.862	3.157
Pregan-20-one	0.000	0.000	0.000	0.000	0.006	0.000	0.000	0.000	0.000	0.000
Pregn-4-ene-3,20-dione	0.000	0.000	0.004	0.016	0.288	0.000	0.000	0.003	0.010	0.032
Stigmasterol	0.000	0.032	0.079	0.156	0.423	0.000	0.017	0.041	0.073	0.127
Tetradecanal	0.021	0.285	0.537	1.316	15.960	0.028	0.226	0.429	0.739	22.813
Tetradecane	0.091	0.252	0.367	0.543	0.902	0.109	0.163	0.287	0.479	0.802
Tetradecene	0.000	0.000	0.000	0.000	0.006	0.000	0.000	0.000	0.000	0.000
Tetratetracontane	0.102	0.256	0.422	0.984	2.604	0.035	0.114	0.256	0.533	1.758
Tocopherol	0.290	9.253	13.029	18.134	42.574	0.000	3.903	8.308	13.417	26.862
Triacontyl acetate	0.000	0.058	0.218	1.282	11.831	0.000	0.026	0.290	1.378	7.037
Undecenoic acid	0.000	0.043	0.195	0.493	1.577	0.000	0.034	0.126	0.318	1.394

Supplementary Table S 5. Compound names and their minimum (Min), 1st quartile (First_qt), median, 3rd quartile (Third_qt) and maximum (Max) relative abundance values detected in femoral pore samples collected from *C. subcristatus* iguanas (yellow) in 2014. Values are separated for males (♂) and females (♀).

	♂					♀				
Compound name	Min	First_qt	Median	Third_qt	Max	Min	First_qt	Median	Third_qt	Max
1-Heneicosanol	0.000	0.000	0.076	0.113	0.521	0.000	0.000	0.120	0.174	0.585
1-Heptadecanol	0.000	0.000	0.000	0.000	0.676	0.000	0.000	0.000	0.000	0.778
1-Hexadecanol	0.000	0.000	0.000	0.030	1.302	0.000	0.000	0.000	0.387	5.590
1-Hexadecen-3-ol, 3,5,11,15-tetramethyl-	0.000	0.000	0.000	0.000	2.600	0.000	0.000	0.000	0.000	0.389
1-Octacosanol	0.000	0.000	0.000	0.000	0.011	0.000	0.000	0.000	0.000	0.017
1-Phenyl-3-(2-phenethyl)hendecane	0.000	0.000	0.000	0.023	0.138	0.000	0.000	0.000	0.021	0.077
1-Tetradecanol	0.000	0.000	0.000	0.000	0.374	0.000	0.000	0.000	0.000	1.533
1-Tridecene	0.000	0.000	0.000	0.000	0.379	0.000	0.000	0.000	0.000	0.000
1,13-Tetradecadien-3-one	0.000	0.000	0.000	0.000	0.000	0.000	0.000	0.000	0.000	0.000
1,16-Hexadecanediol	0.000	0.000	0.000	0.000	0.145	0.000	0.000	0.000	0.000	0.000
1,19-Eicosadiene	0.000	0.000	0.000	0.000	0.000	0.000	0.000	0.010	0.023	0.026
1,37-Octatriacontadiene	0.000	0.000	0.000	0.000	0.242	0.000	0.000	0.000	0.000	0.287
10-Henicosene	0.000	0.000	0.000	0.060	1.302	0.000	0.000	0.050	0.387	0.964
10-Heptadecenoic acid	0.000	0.000	0.139	0.231	0.491	0.000	0.073	0.159	0.262	0.395
10-Methyleicosane	0.088	0.153	0.286	0.401	1.034	0.087	0.127	0.237	0.312	0.594
10-Octadecenoate	0.000	0.014	0.027	0.050	0.088	0.000	0.013	0.072	0.232	0.508
10,12-Pentacosadiynoic acid	0.000	0.000	0.000	0.000	0.000	0.000	0.000	0.000	0.000	0.000
11-Hexadecenal	0.000	0.059	2.498	11.648	26.946	2.239	12.529	15.256	23.449	31.125
11-Hexadecenoic acid	0.000	0.000	0.000	0.006	0.073	0.000	0.000	0.000	0.006	0.129
11-Octadecadien-1-ol	0.000	0.000	0.000	0.000	0.091	0.000	0.000	0.000	0.164	0.467
11-Octadecenoic acid	0.000	0.000	0.014	0.031	0.081	0.000	0.000	0.021	0.198	0.272
12-Docosanoic acid	0.000	0.000	0.000	0.000	0.008	0.000	0.000	0.000	0.000	0.012
12-Methyltetradecanoate	0.000	0.000	0.000	0.000	0.000	0.000	0.000	0.000	0.000	0.000
13-Octadecadien-1-ol	0.000	0.000	0.000	0.020	1.100	0.000	0.000	0.000	0.068	0.275
17-Hydroxypregnenolone	0.000	0.000	0.000	0.001	0.007	0.000	0.000	0.000	0.000	0.005
17-Pentatriacontene	0.000	0.000	0.000	0.035	0.122	0.000	0.000	0.017	0.026	0.177
2-(2-Butynyl)cyclohexanone	0.000	0.000	0.000	0.006	0.024	0.000	0.000	0.001	0.004	0.008
2-Dodecenoic acid	0.000	0.000	0.000	0.000	0.000	0.000	0.000	0.000	0.000	0.003
2-Ethyl-1-dodecanol	0.415	1.421	1.957	2.117	4.597	0.620	1.258	1.623	1.908	3.175
2-Ethylhexyl octanoate	0.000	0.000	0.000	0.000	0.031	0.000	0.000	0.000	0.000	0.314
2-Hepten-4-ol	0.606	1.716	2.083	3.614	9.485	0.000	2.118	3.686	7.991	15.379
2,3-Dimethyloctane	0.000	0.000	0.000	0.135	2.822	0.000	0.000	0.000	0.743	1.970
2,3,3-Trimethyloctane	0.000	0.000	0.000	0.000	0.203	0.000	0.000	0.000	0.000	0.156
2,5,5-Trimethylheptane	0.000	0.116	0.360	0.456	0.645	0.055	0.296	0.368	0.406	0.722
2,6,11-Trimethyldodecane	1.079	1.723	2.517	3.115	5.619	0.640	1.335	2.243	5.106	7.700
2,7,10-Trimethyldodecane	0.520	2.063	2.339	2.901	7.098	0.014	0.020	1.445	2.379	4.524

	♂					♀				
(continues)	Min	First_qt	Median	Third_qt	Max	Min	First_qt	Median	Third_qt	Max
3-Benzyloxy-1,2-diacetyl-1,2-propanediol	0.000	0.112	0.193	0.288	0.635	0.095	0.150	0.177	0.243	0.742
3-Octadecene	0.000	0.000	0.000	0.000	0.321	0.000	0.000	0.000	0.000	0.000
3,4,5,6-Tetramethyloctane	0.000	0.905	1.236	1.932	5.226	0.836	1.044	1.326	1.508	2.783
3,7,11,15-Tetramethyl-2-hexadecen-1-ol	0.000	0.000	0.000	0.223	0.578	0.000	0.000	0.000	0.193	0.235
3,8-Dimethylundecane	0.436	0.964	1.440	1.837	2.083	0.766	0.862	1.218	1.498	5.815
5-Methyl-1-heptanol	0.000	0.000	0.000	0.000	0.000	0.000	0.000	0.000	0.000	0.000
5-Methyl-5-propylnonane	0.595	0.905	1.092	1.834	5.067	0.699	0.986	1.183	1.724	2.455
7-Tetradecenal	0.000	0.000	0.000	0.000	0.009	0.000	0.000	0.000	0.003	0.004
8-Hexadecanoic acid	0.000	0.000	0.000	0.024	0.122	0.000	0.000	0.005	0.025	0.110
8,11,14-Docosatrienoic acid	0.000	0.000	0.000	0.000	0.073	0.000	0.000	0.000	0.152	1.197
9-Hexadecenal	0.000	0.115	0.244	0.506	0.708	0.004	0.047	0.295	0.545	1.891
9-Hexadecenoic acid	0.020	0.042	0.878	1.220	2.901	0.022	0.031	0.054	1.114	2.083
9-Icosene	0.000	0.000	0.000	0.000	0.143	0.000	0.000	0.000	0.000	0.000
9-Octadecene	0.477	1.502	1.897	3.853	24.600	0.478	0.923	1.236	1.582	2.914
9-Tetradecenoic acid	0.000	0.000	0.000	0.000	0.000	0.000	0.000	0.000	0.000	0.000
Alanine	0.000	0.000	0.000	0.000	0.000	0.000	0.000	0.000	0.000	0.000
Androsta-4,16-dien-3-one	0.000	0.000	0.000	0.000	0.101	0.000	0.000	0.000	0.000	0.017
Benzoquinone, 3,5-di-tert-butyl-	0.000	0.000	0.000	0.000	0.000	0.000	0.000	0.000	0.000	0.000
Benzyl icosanoate	0.000	0.000	0.000	0.000	0.000	0.000	0.000	0.000	0.000	0.000
Butenoic acid	0.227	0.363	0.417	0.643	0.769	0.297	0.398	0.497	0.531	1.139
Campesterol	0.000	0.000	0.000	0.000	0.021	0.000	0.000	0.000	0.000	0.000
Cholest-4-en-3-one	0.000	0.000	0.000	0.004	0.010	0.000	0.000	0.001	0.006	0.013
Cholesta-3,5-diene	0.000	0.000	0.000	0.000	9.926	0.000	0.000	0.000	0.000	3.546
Cholestan-3,22,26-triol	0.000	0.000	0.000	0.000	0.000	0.000	0.000	0.000	0.000	0.000
Cholestanol	0.000	0.000	0.000	0.000	0.000	0.000	0.000	0.000	0.000	0.000
Cholesterol	0.000	0.000	0.000	0.000	0.020	0.000	0.000	0.000	0.000	0.091
Crocetane	0.318	0.622	0.799	0.998	1.455	0.347	0.688	0.915	1.407	2.635
Decadiene	0.000	0.000	0.000	0.000	0.004	0.000	0.000	0.000	0.000	0.002
Decane, 5-ethyl-5-methyl-	0.024	0.081	0.145	0.218	0.643	0.045	0.098	0.250	1.090	1.586
Dimethylcapramide	0.000	0.000	0.000	0.000	0.000	0.000	0.000	0.000	0.000	0.000
Dodecane	0.244	0.771	1.078	1.428	3.646	0.210	0.274	0.465	0.861	1.156
Dodecanoic acid	0.000	0.000	0.000	0.000	0.000	0.000	0.000	0.000	0.000	0.000
Eicosane	1.373	4.175	5.664	8.074	10.198	1.412	4.837	5.252	6.679	11.626
Eicosanoic acid	0.000	0.000	0.000	0.000	0.000	0.000	0.000	0.000	0.000	0.152
Elaidyl alcohol	0.000	0.000	0.000	0.000	0.016	0.000	0.000	0.000	0.000	0.000
Ergost-5-en-3-ol	0.000	0.000	0.000	0.000	0.000	0.000	0.000	0.000	0.000	0.000
Ergost-7-en-3-ol	0.000	0.000	0.000	0.000	0.010	0.000	0.000	0.000	0.000	0.006
Erucylamide	0.000	0.000	0.000	0.000	0.006	0.000	0.000	0.000	0.000	0.028
Ethane, 1,2-bis(9-octadecenyloxy)	0.000	0.000	0.000	0.000	0.091	0.000	0.000	0.000	0.000	0.275

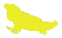

(continues)	♂ Min	♂ First_qt	♂ Median	♂ Third_qt	♂ Max	♀ Min	♀ First_qt	♀ Median	♀ Third_qt	♀ Max
Ethyl docosanoate	0.000	0.000	0.000	0.000	5.323	0.000	0.000	0.000	0.000	10.712
Heptadecyl heptadecanoate	0.200	0.242	0.409	0.759	1.444	0.000	0.377	0.578	0.915	2.596
Hexadecanal	0.156	0.562	2.983	4.496	11.406	0.211	0.373	0.591	3.850	7.771
Hexadecane	0.014	2.088	3.735	5.099	7.214	0.014	0.020	0.103	3.934	11.201
Hexadecanethiol	0.000	0.060	0.100	0.161	0.346	0.000	0.064	0.091	0.112	0.179
Hexadecenoic acid	0.000	0.033	0.396	1.601	4.169	0.026	0.782	1.364	5.849	14.968
Malonic acid	0.000	0.000	0.000	0.000	0.000	0.000	0.000	0.000	0.000	0.000
Methyl 14-methylpentadecanoate	0.000	0.000	0.000	0.000	0.103	0.000	0.000	0.000	0.031	0.780
Methyl 18-methylnonadecanoate	0.000	0.000	0.000	0.000	0.000	0.000	0.000	0.000	0.000	0.000
Methyl 3-hydroxyoctadecanoate	0.000	0.000	0.000	0.000	0.160	0.000	0.000	0.000	0.000	0.096
Methyl hexacosanoate	0.000	0.000	0.000	0.000	0.000	0.000	0.000	0.000	0.000	0.067
n-Hexatriacontane	0.208	0.575	0.996	1.455	2.876	0.271	0.435	0.806	1.107	2.635
n-Tridecanol	0.000	0.000	0.000	0.000	1.708	0.000	0.000	0.000	0.547	7.211
Neopentyl benzoate	0.000	0.000	0.000	0.000	0.000	0.000	0.000	0.000	0.000	0.000
Nonadecanoic acid	0.000	0.000	0.000	0.000	0.000	0.000	0.000	0.000	0.000	0.000
Nonahexacontanoic acid	0.175	0.449	0.822	1.720	2.115	0.171	0.318	0.706	0.891	2.584
Nonanal diethyl acetal	0.000	0.000	0.000	0.000	1.046	0.000	0.000	0.000	0.516	1.264
Octacosyl acetate	0.044	0.132	0.231	0.415	0.949	0.000	0.176	0.234	0.282	0.657
Octadecanal	0.000	0.864	8.819	12.946	16.033	0.000	0.000	0.000	1.723	18.025
Octadecanoic acid	0.000	0.000	0.000	0.000	0.021	0.000	0.000	0.000	0.000	0.076
Octadecedien-1-ol	0.000	0.000	0.000	0.000	0.009	0.000	0.000	0.000	0.000	0.000
Olealdehyde	0.139	10.808	17.090	21.459	50.824	0.080	8.505	16.821	27.679	36.488
Oleyl Alcohol	0.000	0.000	1.160	2.797	5.266	0.000	0.000	0.101	1.093	2.018
Pentadecanal-	0.000	0.000	0.000	0.000	1.923	0.000	0.000	0.000	0.000	0.000
Pentadecane	0.056	0.575	1.246	1.675	2.265	0.157	0.335	1.148	1.648	3.239
Pentadecanoic acid	0.000	0.000	0.000	0.000	0.000	0.000	0.000	0.000	0.000	0.752
Pentanedioic acid	0.000	0.000	0.000	0.000	0.000	0.000	0.000	0.000	0.000	0.000
Pregan-20-one	0.000	0.000	0.000	0.000	0.000	0.000	0.000	0.000	0.000	0.000
Pregn-4-ene-3,20-dione	0.000	0.000	0.021	0.087	0.772	0.000	0.000	0.042	0.151	0.360
Stigmasterol	0.000	0.000	0.000	0.000	0.003	0.000	0.000	0.000	0.000	0.050
Tetradecanal	0.000	4.404	12.269	16.222	19.458	0.000	0.000	0.000	13.880	20.270
Tetradecane	0.777	1.398	1.626	2.151	3.165	0.838	1.409	1.481	1.845	2.755
Tetradecene	0.015	0.073	0.121	0.171	0.446	0.040	0.049	0.142	0.265	0.526
Tetratetracontane	0.234	0.354	0.741	0.838	2.032	0.224	0.426	0.805	1.394	2.190
Tocopherol	0.000	0.000	0.000	0.000	0.000	0.000	0.000	0.000	0.000	0.000
Triacontyl acetate	0.000	0.000	0.000	0.010	0.047	0.000	0.000	0.000	0.005	0.110
Undecenoic acid	0.000	0.000	0.000	0.000	0.000	0.000	0.000	0.000	0.000	0.000

Supplementary Table S 6. Compound names and their minimum (Min), 1st quartile (First_qt), median, 3rd quartile (Third_qt) and maximum (Max) relative abundance values detected in femoral pore samples collected from *C. subcristatus* iguanas (yellow) in 2015. Values are shown only for males (♂) because no females of this species were captured in 2015.

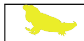	♂				
Compound name	Min	First_qt	Median	Third_qt	Max
1-Heneicosanol	0.000	0.912	1.549	2.914	3.112
1-Heptadecanol	0.000	0.000	0.068	1.890	2.711
1-Hexadecanol	0.000	0.000	0.104	0.196	0.255
1-Hexadecen-3-ol, 3,5,11,15-tetramethyl-	0.000	0.000	0.000	0.000	0.707
1-Octacosanol	0.000	0.003	0.038	0.055	0.103
1-Phenyl-3-(2-phenethyl)hendecane	0.000	0.000	0.000	0.000	0.000
1-Tetradecanol	0.000	0.383	0.400	0.499	0.654
1-Tridecene	0.000	0.000	0.000	0.000	0.000
1,13-Tetradecadien-3-one	0.000	0.000	0.000	0.000	0.002
1,16-Hexadecanediol	0.000	0.155	0.198	0.322	0.333
1,19-Eicosadiene	0.000	0.000	0.000	0.000	0.034
1,37-Octatriacontadiene	0.157	0.192	0.549	1.581	2.718
10-Henicosene	0.000	0.000	0.000	0.000	0.454
10-Heptadecenoic acid	4.161	6.083	7.793	9.020	18.501
10-Methyleicosane	0.098	0.157	0.176	0.212	4.076
10-Octadecenoate	0.627	1.004	1.542	3.425	5.701
10,12-Pentacosadiynoic acid	0.000	0.000	0.000	0.000	0.000
11-Hexadecenal	0.941	1.225	1.750	1.982	2.536
11-Hexadecenoic acid	0.000	0.000	0.053	0.158	0.480
11-Octadecadien-1-ol	0.000	0.122	0.278	0.365	1.191
11-Octadecenoic acid	0.471	0.515	0.663	1.484	5.701
12-Docosanoic acid	0.004	0.038	0.075	0.100	0.175
12-Methyltetradecanoate	0.000	0.000	0.000	0.106	0.936
13-Octadecadien-1-ol	0.155	0.235	0.333	0.537	0.691
17-Hydroxypregnenolone	0.000	0.000	0.000	0.000	0.091
17-Pentatriacontene	0.000	0.000	0.008	0.053	0.207
2-(2-Butynyl)cyclohexanone	0.000	0.000	0.001	0.007	0.010
2-Dodecenoic acid	0.000	0.000	0.000	0.000	0.004
2-Ethyl-1-dodecanol	0.000	0.000	0.000	0.487	8.094
2-Ethylhexyl octanoate	0.000	0.000	0.000	0.000	0.108
2-Hepten-4-ol	0.106	0.164	1.909	2.814	3.395
2,3-Dimethyloctane	0.275	0.340	0.407	0.428	0.608
2,3,3-Trimethyloctane	0.000	0.000	0.000	0.294	0.667
2,5,5-Trimethylheptane	0.065	0.131	0.141	0.169	0.192
2,6,11-Trimethyldodecane	0.000	0.566	1.695	2.532	3.353

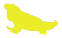	♂				
(continues)	Min	First_qt	Median	Third_qt	Max
2,7,10-Trimethyldodecane	0.022	0.029	0.035	0.036	0.850
3-Benzyloxy-1,2-diacetyl-1,2-propanediol	0.044	0.067	0.086	0.128	0.240
3-Octadecene	0.639	1.089	3.696	3.945	5.665
3,4,5,6-Tetramethyloctane	0.217	0.380	0.469	0.750	0.845
3,7,11,15-Tetramethyl-2-hexadecen-1-ol	0.000	0.000	0.000	0.087	0.190
3,8-Dimethylundecane	0.243	0.313	0.379	0.499	0.632
5-Methyl-1-heptanol	0.000	0.000	0.000	0.000	0.000
5-Methyl-5-propylnonane	0.494	0.539	0.900	1.590	2.894
7-Tetradecenal	0.000	0.000	0.000	0.023	0.033
8-Hexadecanoic acid	0.019	0.051	0.053	0.063	0.103
8,11,14-Docosatrienoic acid	0.000	0.000	0.000	2.543	3.913
9-Hexadecenal	0.071	0.246	0.540	0.880	1.794
9-Hexadecenoic acid	1.913	6.763	10.763	11.411	14.060
9-Icosene	0.000	0.000	0.000	0.000	9.094
9-Octadecene	0.372	2.522	3.529	3.546	4.175
9-Tetradecenoic acid	0.000	0.000	0.000	0.000	0.002
Alanine	0.000	0.000	0.000	0.000	0.000
Androsta-4,16-dien-3-one	0.000	0.000	0.000	0.000	0.032
Benzoquinone, 3,5-di-tert-butyl-	0.000	0.000	0.000	0.000	0.000
Benzyl icosanoate	0.000	0.000	0.000	0.000	0.314
Butenoic acid	0.213	0.266	0.328	0.363	0.382
Campesterol	0.000	0.000	0.000	0.000	0.000
Cholest-4-en-3-one	0.000	0.000	0.000	0.000	0.000
Cholesta-3,5-diene	0.311	0.535	0.567	1.436	1.861
Cholestan-3,22,26-triol	0.000	0.000	0.000	0.000	0.000
Cholestanol	0.000	0.000	0.000	0.000	0.000
Cholesterol	0.000	0.000	0.000	0.000	0.000
Crocetane	0.272	0.383	0.455	0.525	0.968
Decadiene	0.000	0.000	0.000	0.000	0.004
Decane, 5-ethyl-5-methyl-	0.000	0.282	0.368	0.540	0.646
Dimethylcapramide	0.218	0.382	0.533	0.682	1.590
Dodecane	0.108	0.256	0.473	0.500	1.163
Dodecanoic acid	0.000	0.000	0.000	0.000	0.013
Eicosane	0.000	0.000	0.444	0.876	1.071
Eicosanoic acid	0.000	0.000	0.000	0.000	0.000
Elaidyl alcohol	0.000	0.000	0.000	0.025	0.286
Ergost-5-en-3-ol	0.000	0.000	0.000	0.000	0.014
Ergost-7-en-3-ol	0.000	0.174	0.439	0.481	0.781
Erucylamide	0.000	0.000	0.000	0.000	0.000

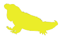	♂				
(continues)	Min	First_qt	Median	Third_qt	Max
Ethane, 1,2-bis(9-octadecenyloxy)	0.183	1.167	3.092	3.224	3.846
Ethyl docosanoate	0.000	0.000	0.000	0.000	0.000
Heptadecyl heptadecanoate	0.000	0.046	0.391	8.472	11.394
Hexadecanal	0.000	0.051	0.092	0.645	0.766
Hexadecane	0.036	0.629	0.724	0.856	1.089
Hexadecanethiol	0.000	0.000	0.000	0.036	0.977
Hexadecenoic acid	0.221	0.881	2.467	4.904	7.019
Malonic acid	0.000	0.000	0.000	0.000	0.000
Methyl 14-methylpentadecanoate	0.416	3.115	3.342	5.499	6.326
Methyl 18-methylnonadecanoate	0.047	0.697	3.688	6.741	9.887
Methyl 3-hydroxyoctadecanoate	0.000	0.000	0.000	0.000	0.000
Methyl hexacosanoate	0.151	5.640	6.811	7.731	9.276
n-Hexatriacontane	0.272	0.277	0.370	0.455	0.746
n-Tridecanol	0.000	0.952	2.365	22.601	35.996
Neopentyl benzoate	0.000	0.000	0.000	0.000	0.000
Nonadecanoic acid	0.000	0.000	0.000	0.000	0.000
Nonahexacontanoic acid	0.000	0.270	0.329	0.396	0.587
Nonanal diethyl acetal	0.000	0.000	0.000	0.000	0.000
Octacosyl acetate	0.000	0.000	0.127	0.167	0.758
Octadecanal	0.000	0.000	0.136	2.705	3.197
Octadecanoic acid	0.000	0.000	0.000	0.000	0.205
Octadecedien-1-ol	0.000	0.000	0.000	0.000	0.025
Olealdehyde	0.388	0.629	3.119	3.413	16.381
Oleyl Alcohol	0.000	0.000	0.000	0.000	0.000
Pentadecanal-	0.000	0.000	0.000	0.597	0.825
Pentadecane	0.000	0.000	0.044	0.424	4.109
Pentadecanoic acid	0.000	0.000	0.000	0.000	0.000
Pentanedioic acid	0.000	0.000	0.000	0.000	0.000
Pregan-20-one	0.000	0.000	0.000	0.000	0.000
Pregn-4-ene-3,20-dione	0.000	0.148	1.206	2.682	3.040
Stigmasterol	0.000	0.000	0.002	0.019	0.037
Tetradecanal	0.000	0.000	0.000	0.192	0.801
Tetradecane	0.572	0.752	0.850	0.988	3.898
Tetradecene	0.038	0.063	0.077	0.085	0.091
Tetratetracontane	0.047	0.134	0.214	0.298	6.674
Tocopherol	0.000	0.000	0.000	0.000	0.000
Triacontyl acetate	0.029	0.051	0.073	0.083	0.112
Undecenoic acid	0.000	0.000	0.000	0.071	0.159

Supplementary Figures

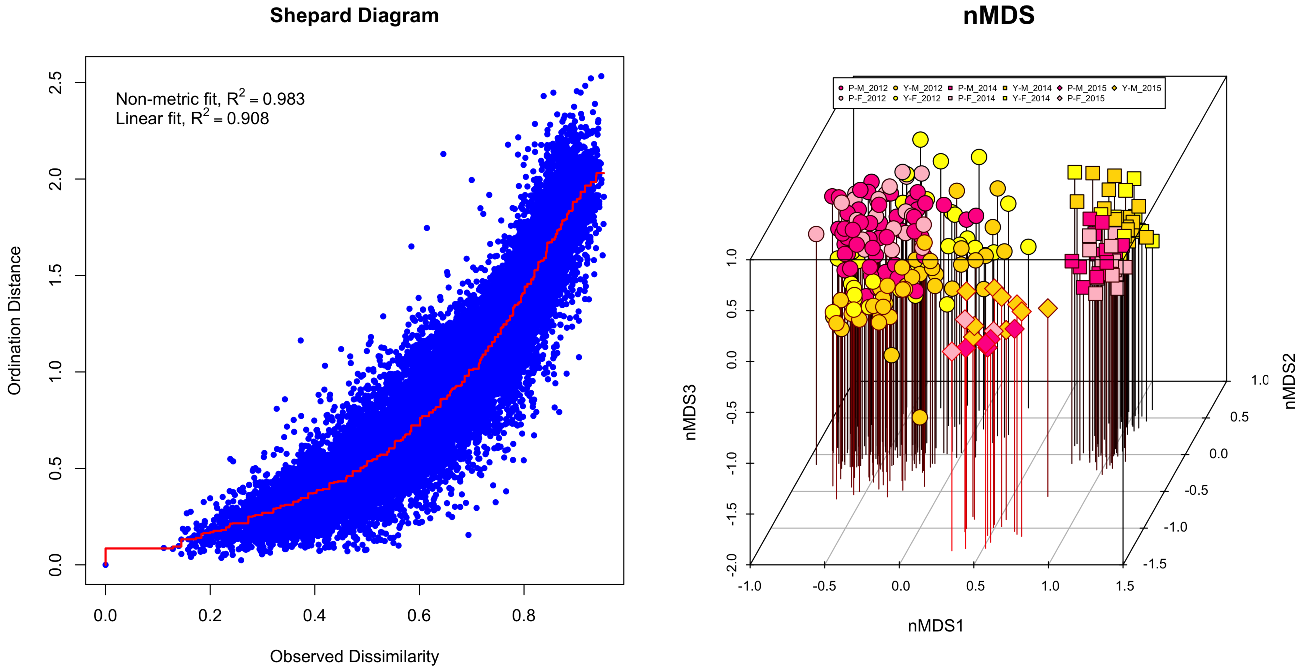

Supplementary Figure S 1. Left : Shepard diagram for non-metric multidimensional scaling (nMDS). This diagram illustrates the goodness of fit for nMDS data reduction. While some of the information is lost during data scaling, the quality of the ordination is still fair after the transformation. **Right**: Three dimensional nMDS plot. Samples are identified by year, species and sex (circles = 2012, squares = 2014, diamonds = 2015; dark-pink = male *C. marthae*, light-pink = female *C. marthae*, dark-yellow = male *C. subcristatus*, light-yellow = female *C. subcristatus*). The lines colored in red highlight data in foreground as compared to the ones in the background (black lines).

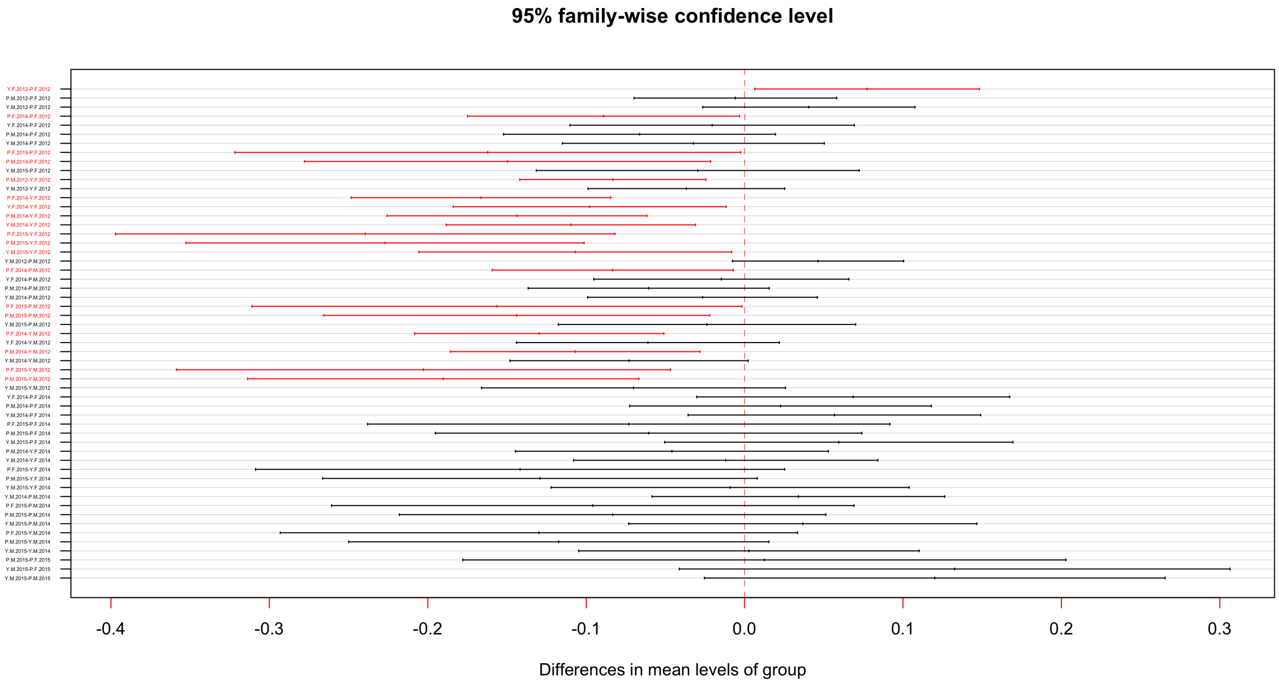

Supplementary Figure S 2. We used a Tukey's HSD analysis on multivariate spread to test for significant differences between groups. The pairwise comparisons for which multivariate spread were significantly different are represented in red.

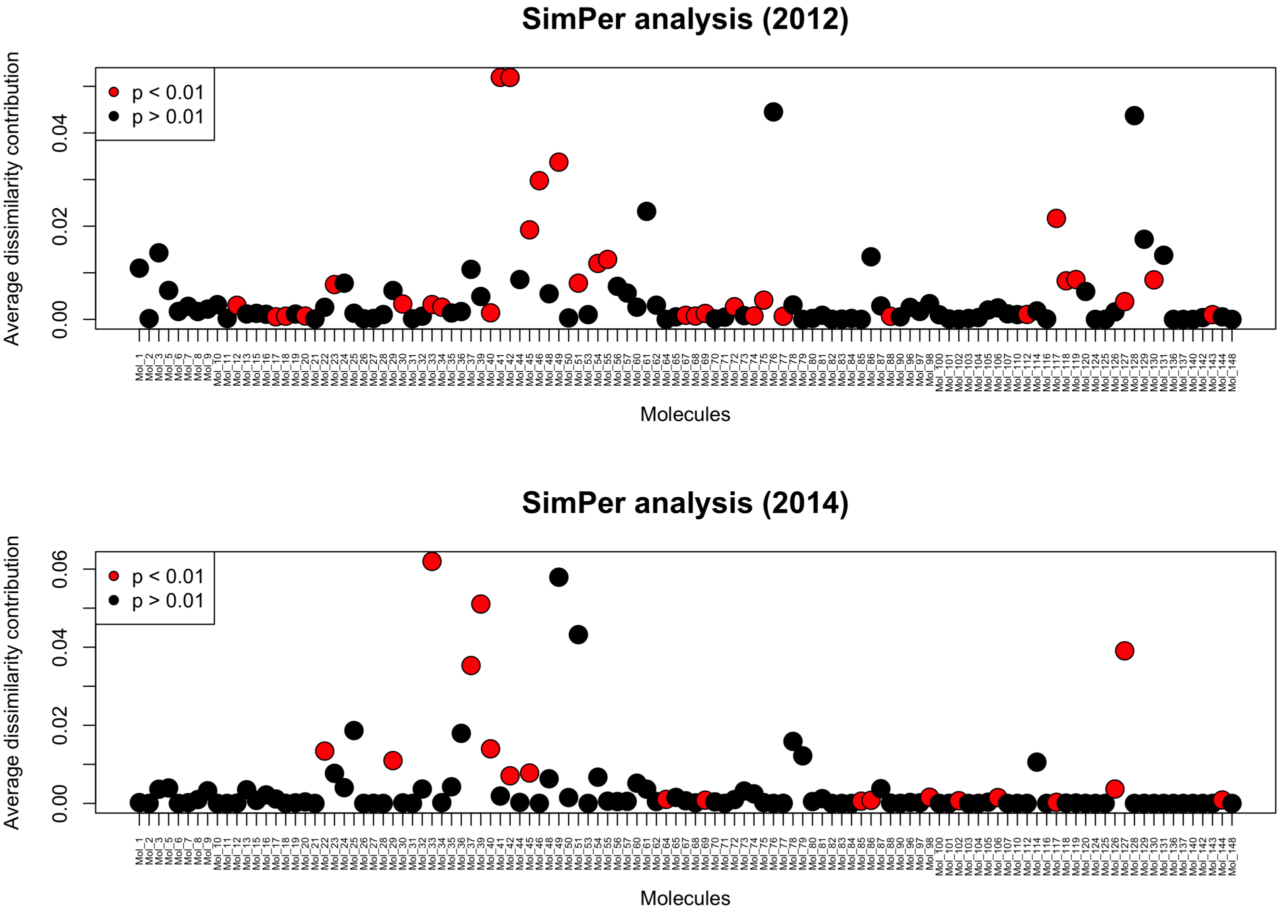

Supplementary Figure S 3. Results of SIMPER analysis. Red dots represents molecules deemed significant in determining dissimilarity patterns between species in the two reproductive seasons (2012 and 2014).

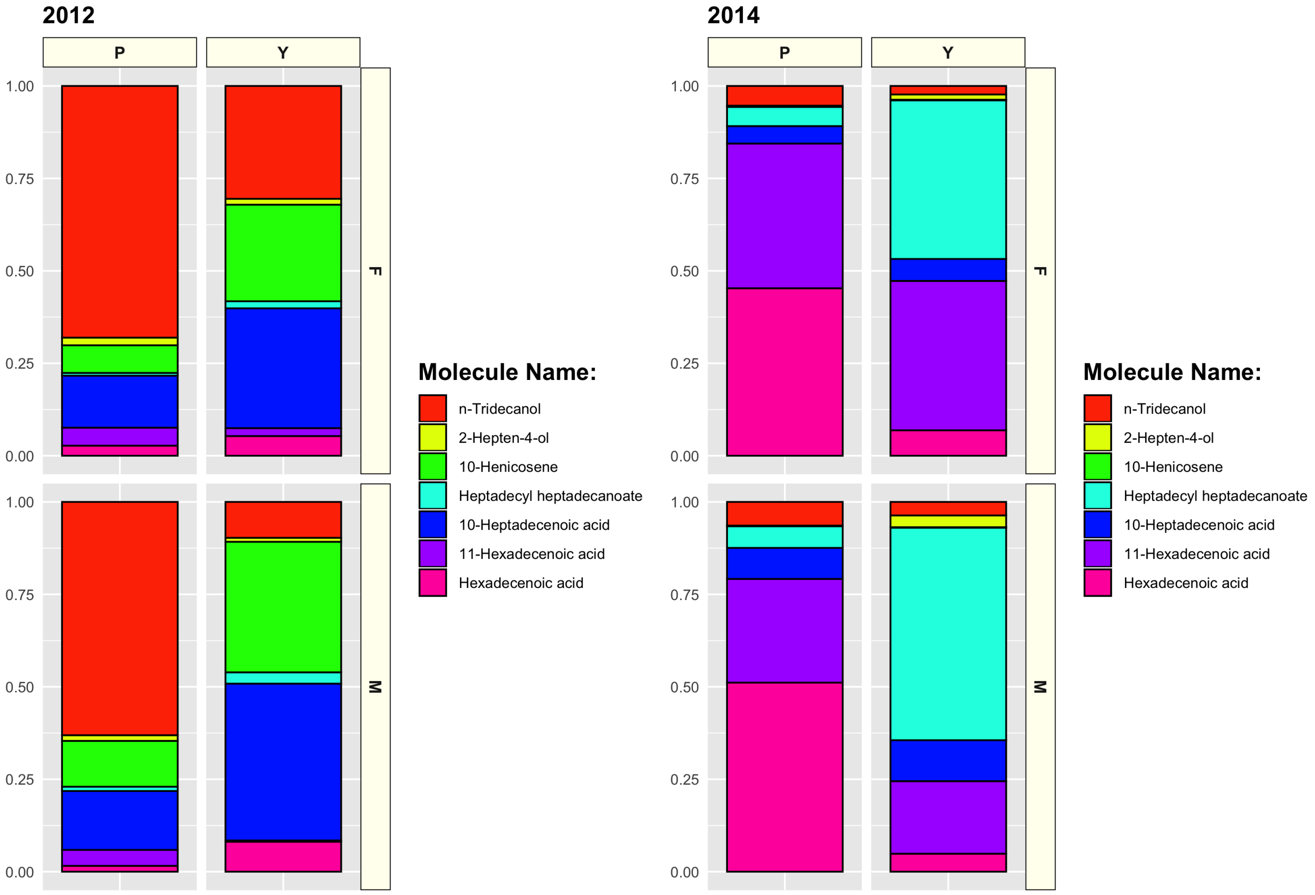

Supplementary Figure S 4. Relative abundance of different molecules identified by year (2012; 2014) in the SIMPER analysis. Data are organized by species (P = *C. marthae*; Y = *C. subcristatus*) and sex (M = Males; F = Females).

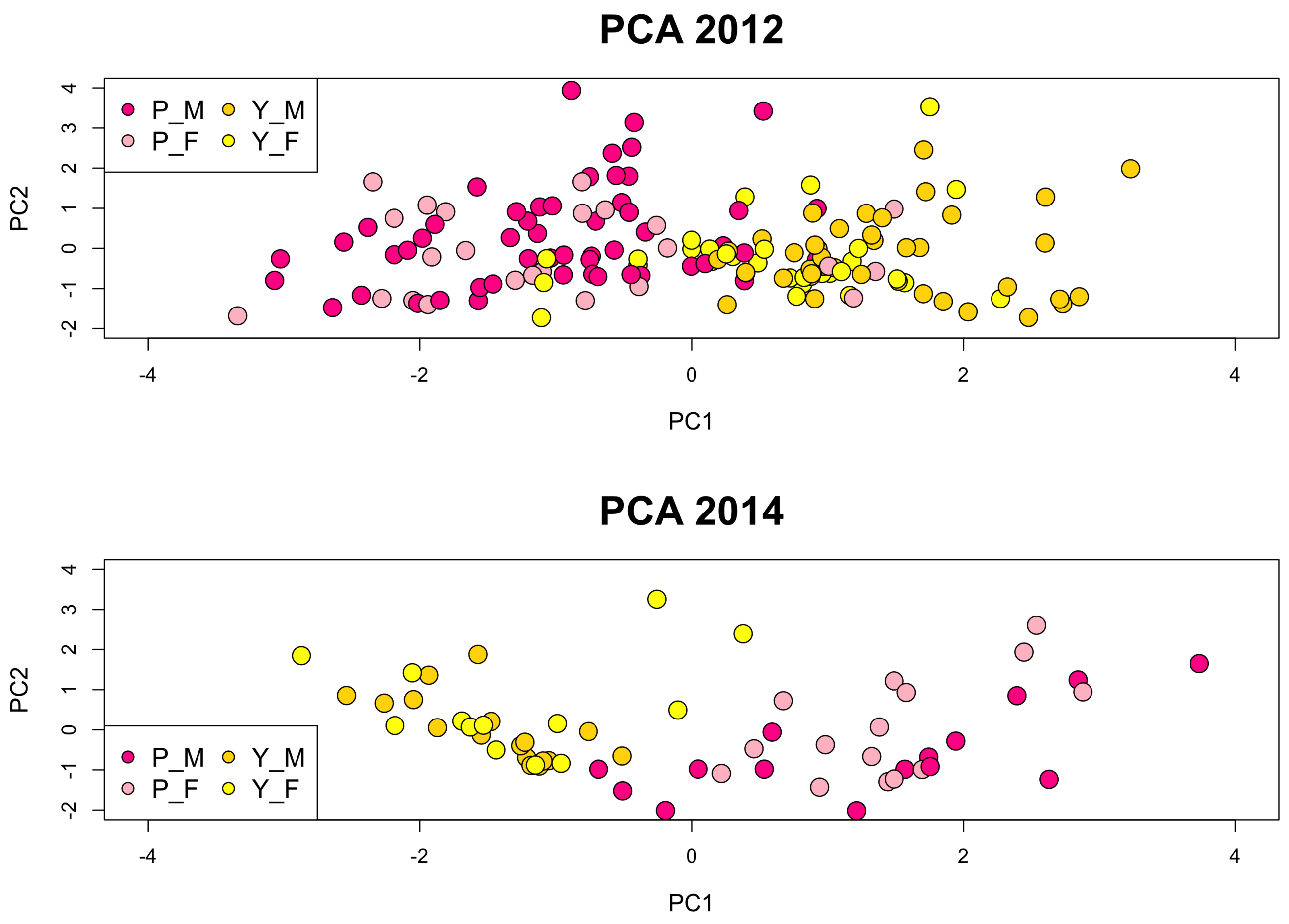

Supplementary Figure S 5. Principal component analysis built using the 7 molecular species driving the differentiation of species in the two reproductive seasons (2012 and 2014). Every dot represents an individual and is colored according to iguana species and sex. (P-M = Pink Males, P-F = Pink Females, Y-M = Yellow-Males, Y-F = Yellow Females)

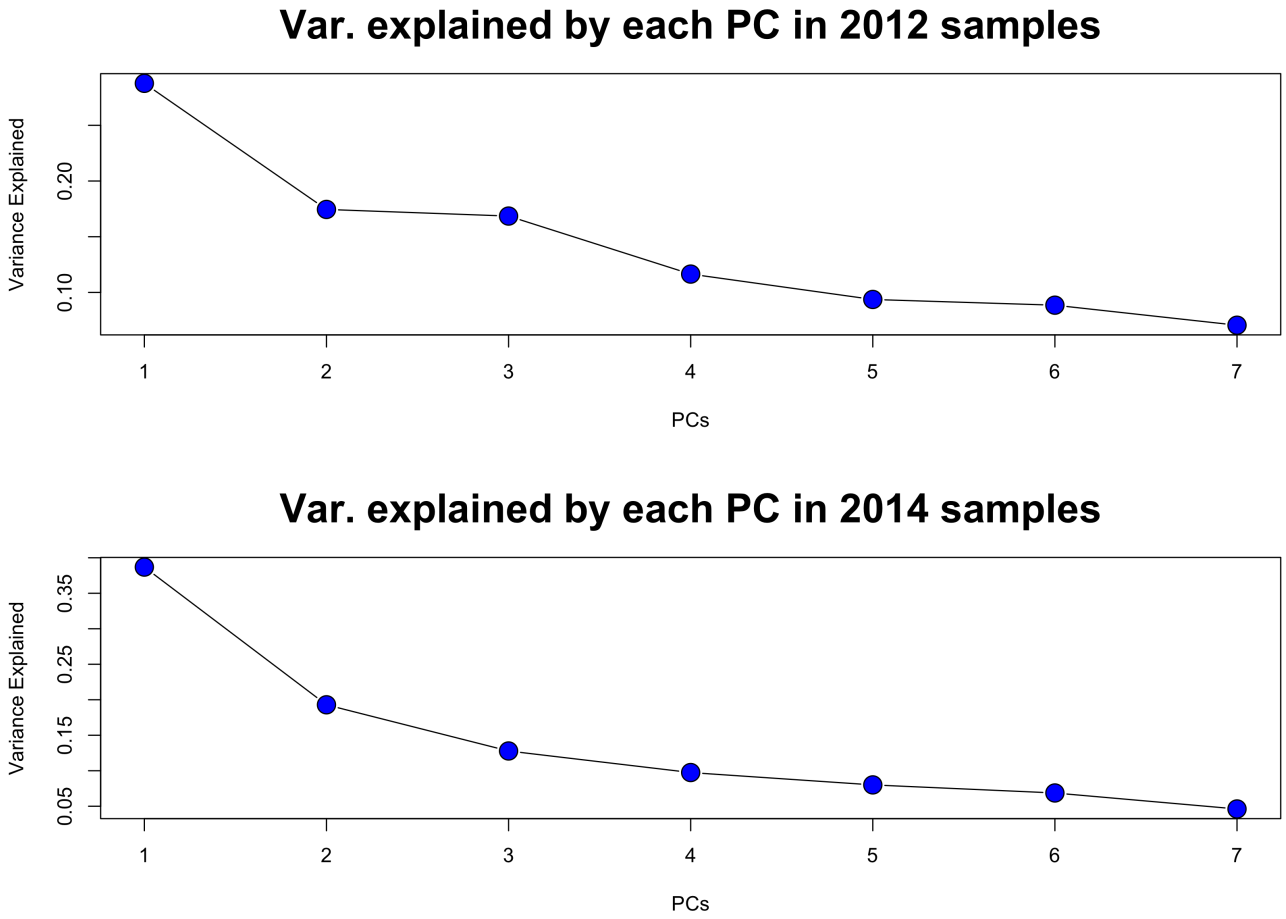

Supplementary Figure S 6. Variance (Var.) explained by each principal component (PC) in the two reproductive seasons (2012 and 2014). The loading value of the principal component explaining the largest variance was associated with 10-Henicosene compound in both years.

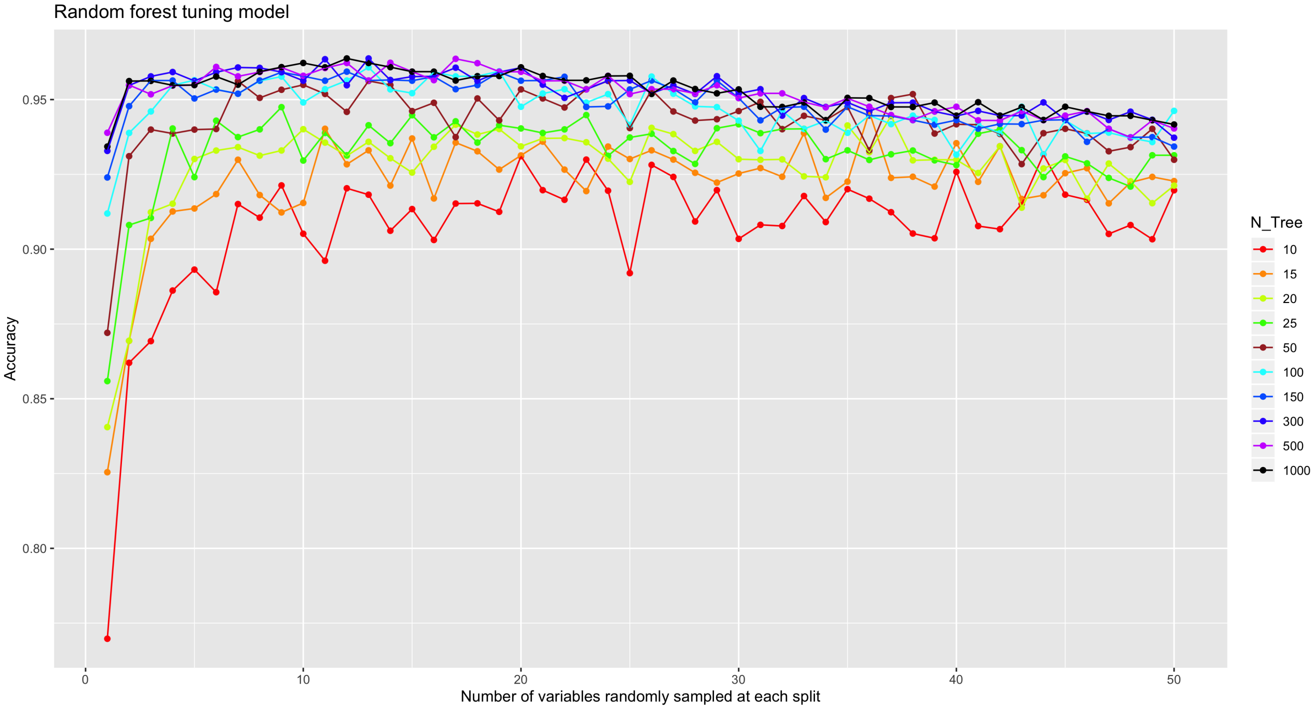

Supplementary Figure S 7. Random Forest tuning model. We tested different values of number of trees to grow (ntree), and number of variables (chemical compounds) to be randomly selected at each branching of the tree. Growing a model with 500 trees and 17 variables at each split yielded the highest accuracy.

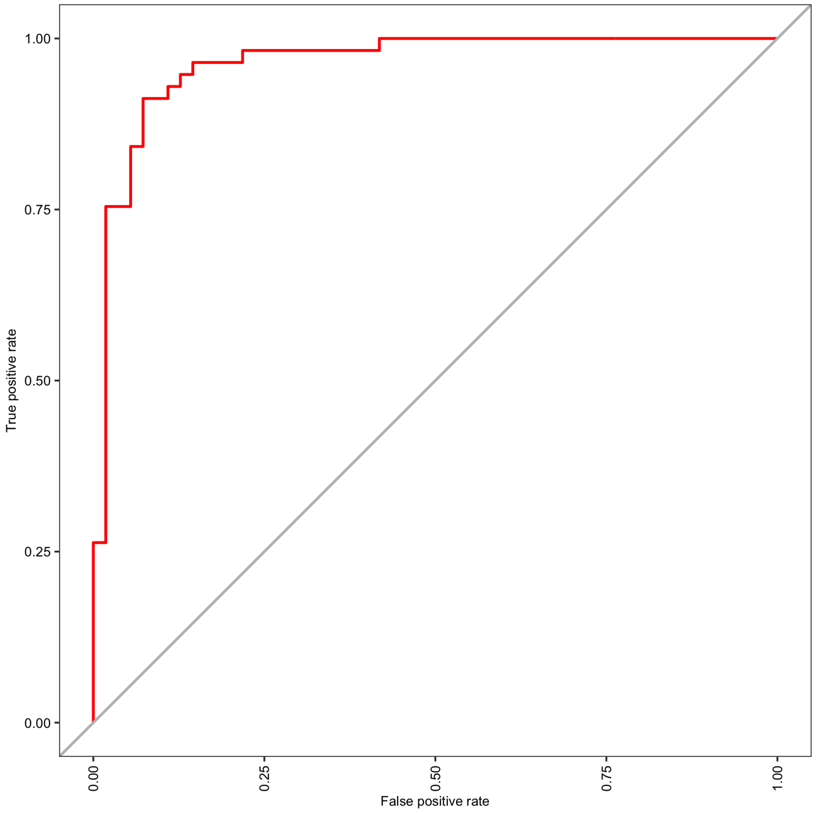

Supplementary Figure S 8. Receiving-Operating-Characteristic (ROC) curve. The Area Under the ROC curve, shown in red, is estimated at 96% of the true positive rate space, indicating that our model performs much better than random predictions (represented by the diagonal line).
